# The person-to-person transmission landscape of the gut and oral microbiomes

**DOI:** 10.1038/s41586-022-05620-1

**Published:** 2023-01-18

**Authors:** Mireia Valles-Colomer, Aitor Blanco-Míguez, Paolo Manghi, Francesco Asnicar, Leonard Dubois, Davide Golzato, Federica Armanini, Fabio Cumbo, Kun D. Huang, Serena Manara, Giulia Masetti, Federica Pinto, Elisa Piperni, Michal Punčochář, Liviana Ricci, Moreno Zolfo, Olivia Farrant, Adriana Goncalves, Marta Selma-Royo, Ana G. Binetti, Jimmy E. Becerra, Bei Han, John Lusingu, John Amuasi, Loredana Amoroso, Alessia Visconti, Claire M. Steves, Mario Falchi, Michele Filosi, Adrian Tett, Anna Last, Qian Xu, Nan Qin, Huanlong Qin, Jürgen May, Daniel Eibach, Maria Valeria Corrias, Mirco Ponzoni, Edoardo Pasolli, Tim D. Spector, Enrico Domenici, Maria Carmen Collado, Nicola Segata

**Affiliations:** 1 Department CIBIO, University of Trento, Trento, Italy; 2 Department of Experimental Oncology, IEO European Institute of Oncology IRCCS, Milan, Italy; 3 Clinical Research Department, Faculty of Infectious and Tropical Diseases, London School of Hygiene and Tropical Medicine, London, UK; 4 Institute of Agrochemistry and Food Technology-National Research Council (IATA-CSIC), Paterna, Valencia, Spain; 5 Instituto de Lactología Industrial (CONICET-UNL), Facultad de Ingeniería Química, Universidad Nacional del Litoral, Santa Fe, Argentina; 6 Grupo de Investigación Alimentación y Comportamiento Humano, Universidad Metropolitana, Barranquilla, Colombia; 7 School of Public Health, Health Science Center, Xi’an Jiaotong University, Xi’an, China; 8 National Institute for Medical Research, Tanga Medical Research Centre, Tanga, Tanzania; 9 Kumasi Centre for Collaborative Research in Tropical Medicine, Kwame Nkrumah University of Science and Technology, Kumasi, Ghana; 10 Oncology Unit, IRCCS Istituto Giannina Gaslini, Genoa, Italy; 11 Department of Twin Research and Genetic Epidemiology, King’s College London, London, UK; 12 Centre for Microbiology and Environmental Systems Science, University of Vienna, Vienna, Austria; 13 Shanghai Tenth People’s Hospital, Tongji University School of Medicine, Shanghai, China; 14 Realbio Genomics Institute, Shanghai, China; 15 Bernhard Nocht Institute for Tropical Medicine, Hamburg, Germany; 16 Laboratory of Experimental Therapies in Oncology, IRCCS Istituto Giannina Gaslini, Genoa, Italy; 17 Department of Agricultural Sciences, University of Naples ‘Federico II’, Portici, Italy; 18 Centre for Computational and Systems Biology (COSBI), Microsoft Research Foundation, Rovereto, Italy

**Keywords:** Genome informatics, Metagenomics, Microbiome, Bacterial genetics

## Abstract

The human microbiome is an integral component of the human body and a co-determinant of several health conditions^1,2^. However, the extent to which interpersonal relations shape the individual genetic makeup of the microbiome and its transmission within and across populations remains largely unknown^3,4^. Here, capitalizing on more than 9,700 human metagenomes and computational strain-level profiling, we detected extensive bacterial strain sharing across individuals (more than 10 million instances) with distinct mother-to-infant, intra-household and intra-population transmission patterns. Mother-to-infant gut microbiome transmission was considerable and stable during infancy (around 50% of the same strains among shared species (strain-sharing rate)) and remained detectable at older ages. By contrast, the transmission of the oral microbiome occurred largely horizontally and was enhanced by the duration of cohabitation. There was substantial strain sharing among cohabiting individuals, with 12% and 32% median strain-sharing rates for the gut and oral microbiomes, and time since cohabitation affected strain sharing more than age or genetics did. Bacterial strain sharing additionally recapitulated host population structures better than species-level profiles did. Finally, distinct taxa appeared as efficient spreaders across transmission modes and were associated with different predicted bacterial phenotypes linked with out-of-host survival capabilities. The extent of microorganism transmission that we describe underscores its relevance in human microbiome studies^5^, especially those on non-infectious, microbiome-associated diseases.

## Abstract (web summary)

Data from more than 9,700 human stool and oral metagenomes has been used to decipher the strain transmission patterns of the human microbiome from mother to infant, within households and within populations.

## Main

Our genome is inherited from our parents and remains stable over our lifetime, with limited accumulation of nucleotide variations. By contrast, the genetic makeup of our microorganism complement (the human microbiome) is seeded at birth and changes over time, displaying both high temporal variability and personalization^6,7^. Factors including diet and lifestyle are well known to modulate the composition of the human microbiome^1,2,8^, but as very few members of the microbiome can thrive outside the human body, most microorganisms must be acquired from other individuals^3,4^. Indeed, colonization of the human gut by microorganisms is largely seeded by maternal transmission^9–14^, but maternal seeding alone cannot account for the large diversity of microorganisms found in adults. How members of the microbiome are acquired and transmitted by individuals and spread in populations, and how this shapes the personal microbiome genetic makeup remain largely unexplored—especially in humans^15,16^—with only preliminary findings to date^11,17^. So far, research has been hindered by the limited number and size of accurately designed studies, and by the difficulties in consistently and comprehensively profiling microorganism conspecific strains—that is, genetic variants within species.

Strains are the individual-specific building blocks of the human microbiome^18,19^. They can be highly genomically and functionally divergent within a species, and their profiling is a necessary prerequisite to distinguish transmission of microorganisms from microbiome convergence towards an overlapping set of species. Identifying the features of microbiome transmission will advance our understanding of the complexity of the human microbiome, and can help address the ‘communicable’ factor that microbiome transmission adds to diseases and conditions currently considered non-communicable^5^. Here, we characterize and quantify the patterns of person-to-person microbiome strain sharing across multiple scenarios to provide a comprehensive description of the microbiome transmission landscape.

## Profiling microbiome transmission

**Fig. 1:**
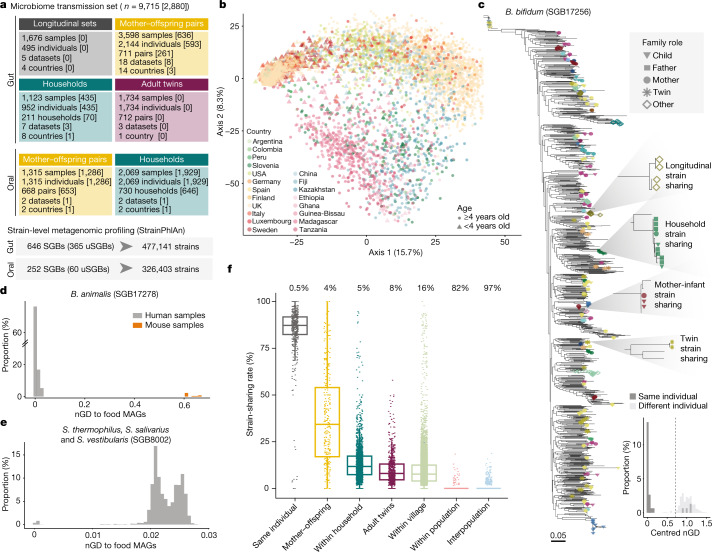
A metagenomic framework to survey person-to-person microbiome strain transmission. **a**, Overview of the study and dataset based on the SGB framework (Methods). Numbers in square brackets are the number of units sequenced in this study. **b**, Overall species-level structure of the gut samples (principal component analysis on Aitchison distance, one random sample per individual, *n* = 4,840). Samples are coloured by country and shapes indicate age. **c**, Phylogeny of *B. bifidum* (SGB17256) (Methods), a low-prevalence highly transmitted species (Supplementary Table 9), showing the genetic diversity of strains and the shared strains between samples of the same individual and between different individuals. One example of strain sharing is highlighted for each relationship type. Tree leaves involved in strain-sharing instances are coloured by dataset (Extended Data Fig. 1b) and their shapes reflect kinship. Bottom, the distribution of pairwise centred nGDs of the species in individuals sampled at two time points (less than six months apart, ‘same individual’) and in unrelated individuals (‘different individual’; Extended Data Fig. 3 and Methods), confirming the suitability of the methodology to infer strain identity. **d**,**e**, The distribution of pairwise nGDs between *B. animalis* (SGB17278) (**d**) and *S. thermophilus*, *S. salivarius* and *S. vestibularis* (SGB8002) (**e**) strains reconstructed from human gut metagenomes or mouse samples and MAGs reconstructed from fermented food^40^. The presence of *B. animalis* in humans is associated with the consumption of commercial dietary products (Extended Data Fig. 4a), whereas only a subset of *S. thermophilus*, *S. salivarius* and *S. vestibularis* strains is associated with fermented food intake (Extended Data Fig. 4b). **f**, Person-to-person strain-sharing rates (number of shared strains/number of shared SGBs × 100%) across relationship types. All comparisons are statistically significant (Kruskal–Wallis test, *n* = 26,218, *χ*^2^ = 11,420, *P* < 2.2 × 10^−16^, post hoc Dunn tests, *P*_adj_ < 0.05; Supplementary Table 7). In box plots, box edges delineate lower and upper quartiles, the centre line represents the median and whiskers extend to 1.5 times the interquartile range (IQR). The number along the top is the percentage of pairs between which no strain-sharing event was detected.

**Extended Data Fig. 1:**
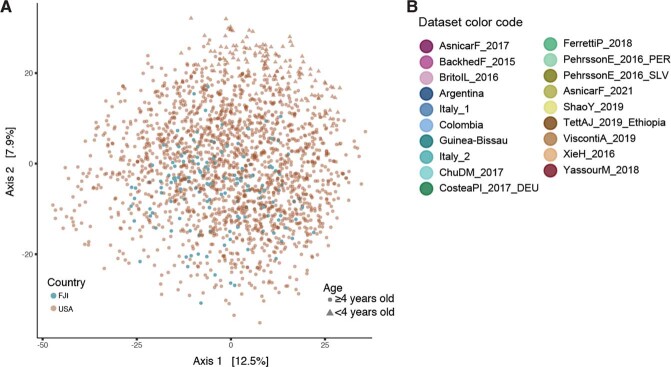
Data overview. **A**) Species-level ordination (PCoA on Aitchison distance, N = 2,069 samples) reflecting the overall microbiome diversity spanned by the oral microbiome samples considered. Samples are coloured by country, while shapes depict age. **B**) Colour code of the samples in the phylogenetic tree in Fig. 1c, representing the datasets they belong to.

To unravel the modes of person-to-person microbiome transmission we performed an integrative analysis on a large set of metagenomic datasets^2,9,10,12,20–34^ with known family relationships (*n* = 31) that were analysed using improved strain-level profiling metagenomic tools (Methods). Eight of these datasets were newly sequenced in the context of this study from different geographical areas and host lifestyles in America (Argentina, Colombia and the USA), Africa (Guinea-Bissau), Asia (China) and Europe (Italy). Three other studies^9,34^ in Africa (Ghana and Tanzania) and Europe (Italy) were further expanded here for a total of 978 stool and 1,929 saliva samples (Supplementary Tables 1 and 2). This collection comprises 9,715 microbiome samples (7,646 stool and 2,069 saliva) and curated host information, enabling the assessment of transmission across mother–infant pairs, household members, adult twin pairs, villages and populations. Although the 31 datasets differ in size, with human metagenomes from 20 different countries in five continents and representing diverse host lifestyles (Fig. 1a,b, Extended Data Fig. 1a and Supplementary Table 2), the integrated set facilitates the identification of person-to-person microbiome transmission patterns at the global level.

**Extended Data Fig. 2:**
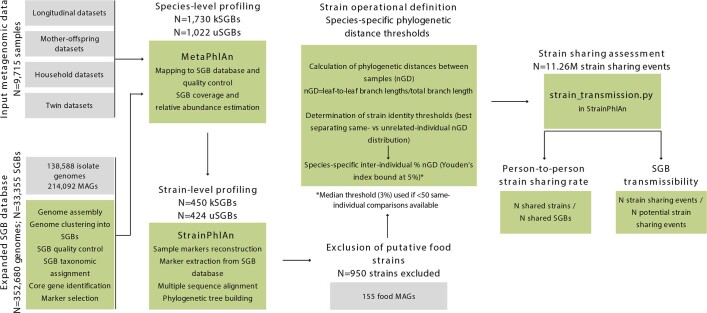
Strain sharing workflow. Workflow used to assess strain sharing in the current manuscript.

**Extended Data Fig. 3:**
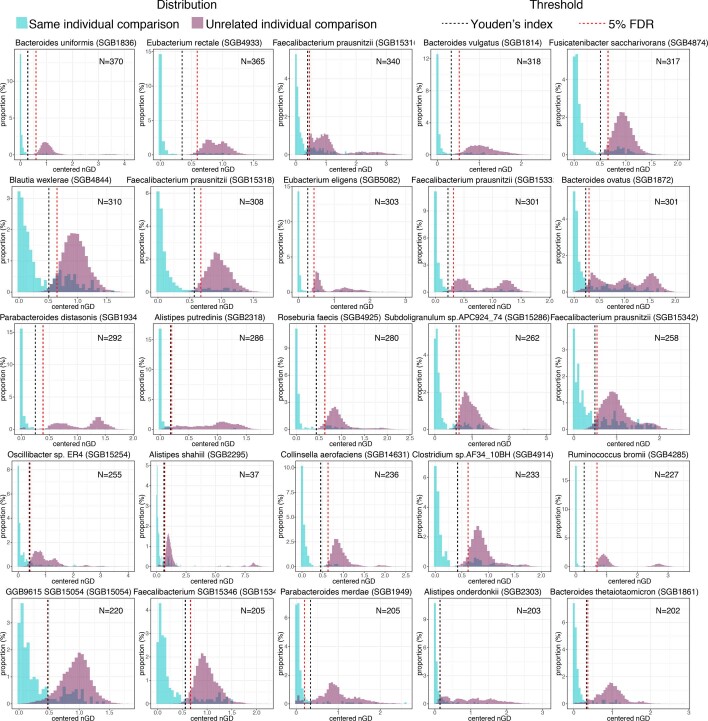
Species-specific operational definitions of strain. Comparison of same-individual (green) to unrelated individual (purple) genetic distance comparisons for the 25 most prevalent SGBs in gut metagenome longitudinal datasets. Strain identity thresholds were set as the Youden’s index (black dashed line) or as the 5th percentile of the unrelated individual comparisons (red dashed line) when the first was above 5% (e.g. *Parabacteroides merdae* [SGB1949]). Centred nGD: normalised phylogenetic distance divided by the median nGD of the phylogenetic tree. The N in each histogram corresponds to the number of same-individual comparisons in which each SGB was profiled at strain-level.

Microorganism strain transmission inference via metagenomics exploits the validated assumption that strains usually persist within an individual’s gut over periods of at least a few months but are rarely found in unrelated individuals unless direct or indirect transmission has occurred^19,35–38^. Here, we first improved our strain-level profiling methodology^39^ (Methods), and then further refined strain tracking with operational species-specific definitions of strain identity (Extended Data Fig. 2). Strain boundaries were set by identifying the normalized phylogenetic distance (nGD) thresholds that best separated same-individual longitudinal strain retention from unrelated individual nGD distributions in more than 1,500 longitudinal samples from 4 countries^20,22,27,28,31^ (Youden’s index allowing <5% potential false positives—that is, same strain shared by unrelated individuals; permutation ANOVA (PERMANOVA), *n* ≥ 50 pairs, *R*^2^ = 0.75 to 1%, *P* < 0.001; Fig. 1c, Extended Data Fig. 3, Supplementary Table 3 and Methods). Such nGD-based thresholds perform well with phylogenies built with the rather low average coverage that is typical for most detectable species in metagenomic samples (mean coverage = 7.2×) and with limited lengths of the concatenated marker gene alignments (mean trimmed alignment length = 74,348 nucleotides (nt)). In addition, our approach exploits the information on evolutionary models that is provided by phylogenetic trees that is not available when considering raw single-nucleotide variation (SNV) rates or genetic similarity.

Microbiome profiling was also expanded to 1,022 not yet cultured and unnamed species (referred to as unknown species-level genome bins (uSGBs)), complementing the 1,730 species with cultured representatives (known species-level genome bins (kSGBs)) defined in a repository of more than 214,000 metagenome-assembled genomes (MAGs) and around 138,000 available isolate genomes^39^. uSGBs constitute 37% of all detected species-level genome bins (SGBs) and were found to be highly prevalent (86% of gut and 100% of oral metagenomes, with 17% and 10% median relative abundance, respectively), especially in gut metagenomes from non-westernized communities (99% prevalence, with 42% median relative abundance overall; Methods). Strain sharing was assessed by profiling in each sample the dominant strain of SGBs found with at least 10% prevalence and in at least 20 samples of at least one cohort, for a total of 646 SGBs in gut metagenomes (Supplementary Table 4) and 252 SGBs in oral metagenomes (Supplementary Table 5), with 24 SGBs profiled in both environments. The developed computational methodology is publicly available for strain-transmission inference from any metagenomic dataset (Methods and Code availability).

As a case in point, *Bifidobacterium bifidum* (SGB17256)—one of the 646 gut SGBs assessed for transmission—was successfully profiled in 1,298 gut microbiome samples (17% of total stool samples). We detected the same *B. bifidum* strain in 87% of pairs of samples from the same individual collected up to six months apart, with nGD between strains following a clear bimodal distribution (the first peak at phylogenetic distance close to zero indicating shared strains) (Fig. 1c). Overall, 13,278 instances of inter-individual shared *B. bifidum* strains were identified between the vast majority of mothers and their offspring (proportion of strain-sharing events detected over potential transmissions—that is, SGB transmissibility = 0.93; Methods) as well as among household members (SGB transmissibility = 0.73).

**Extended Data Fig. 4:**
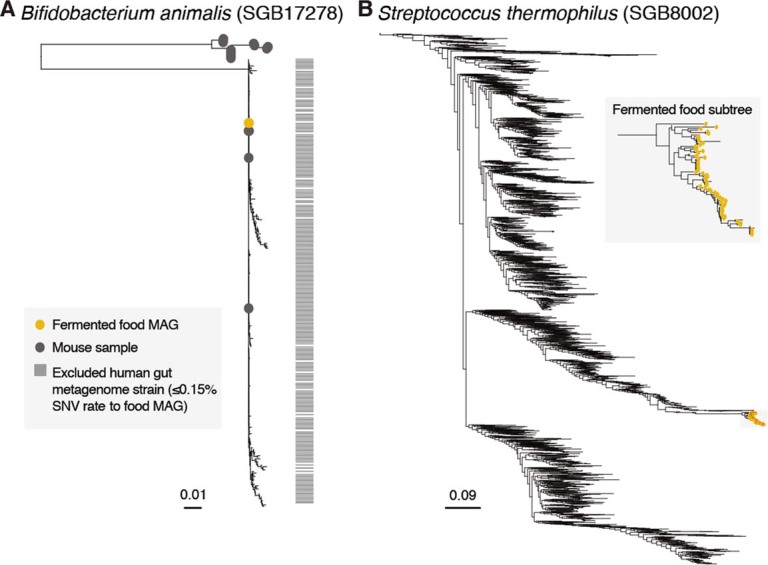
Phylogenetic trees of species containing strains found in food. **A**) Phylogeny of *Bifidobacterium animalis* (SGB17278) produced with StrainPhlAn (*Methods*) including strains reconstructed from human gut metagenomes, from mice samples (grey dots) and MAGs reconstructed from fermented food 32 (yellow dots). Differently from strains found in mice, 94% of human-derived strains are at ≤0.0015 single nucleotide variation (SNV) rate to MAGs obtained from fermented food (*Methods*), suggesting that the presence of this species in humans is associated with consumption of commercial dietary products, and were consequently excluded from further analyses (horizontal grey bars). **B**) Phylogeny of *Streptococcus thermophilus-salivarius-vestibularis* (SGB8002) produced with StrainPhlAn (*Methods*) including strains reconstructed from human gut metagenomes together with MAGs reconstructed from fermented food 32 (yellow dots), suggesting only a subset of strains found in the human gut is associated with fermented food intake. Only the leaves in the enlarged subtree (“Fermented food subtree”) were at ≤0.0015 single nucleotide variation (SNV) rate to MAGs obtained from fermented food (Methods) and were consequently excluded from further analyses.

Even though disentangling direct transmission from indirect acquisition or co-acquisition is possible only with longitudinal sampling or in specific settings (for example, mother to newborn), we minimized the chances of detecting strain sharing resulting from co-acquisition from common dietary sources by identifying and discarding in each SGB those strains with high similarity (≤0.0015 SNV rate) to MAGs or isolate genomes of microorganisms obtained from commercial fermented foods^40^ (Methods). Because food microbiomes remain poorly investigated, other strains or species might originate from food sources even though food-to-gut colonization is regarded as rare^40^. This filtering resulted in the exclusion from the downstream analysis of most *Bifidobacterium animalis* (SGB17278) strains (278 strains, 94% of the total; Fig. 1d, Extended Data Fig. 4a, Supplementary Table 6 and Methods) in gut samples, supporting its putative origin from commercial dietary products^20^. Indeed, more than 98% of excluded samples were from westernized datasets, whereas only 6 strains were detected in non-westernized datasets (less than 0.07% of non-westernized samples), from locations where commercial probiotics are less available. Following the same criterion, 540 strains being phylogenetically close to MAGs of food origin were excluded from 7 other SGBs, including *Streptococcus thermophilus, S. salivarius* and *S. vestibularis* (SGB8002) (19 strains excluded; Fig. 1e, Extended Data Fig. 4b and Supplementary Table 6). Overall, after these exclusions, we detected around 6.35 million instances of strain sharing between different individuals in gut samples and around 4.91 million in oral samples.

## Overview of gut microbiome transmission

We first assessed general gut microbiome strain-sharing patterns across human relationships, defining person-to-person strain-sharing rates as the number of strains shared between two individuals normalized by the number of SGBs profiled in common (out of the 646 SGBs profiled at strain level; Methods). Strains were confirmed to be highly persistent in subjects sampled less than six months apart^20,22,27,28,31^ (median 87% strain-sharing rate), with as little as 0.5% of individuals displaying no longitudinal overlap in the detected strains—potentially owing to the occurrence of unreported perturbations or sample mislabelling. The highest person-to-person strain-sharing rates were detected between cohabiting mothers and their 0- to 3-year-old offspring (median of 34% strain-sharing rate), followed by individuals 4 years of age and older in the same household (12%), non-cohabiting adult twins (8%), and non-cohabiting adults in the same village (8%). Whereas strain sharing between adult twins might in part result from persisting shared maternal transmission, strain sharing among individuals in the same village is probably the result of horizontal transmission through physical interaction and the shared environment. By contrast, non-cohabiting individuals in different villages of the same and of different population-specific study cohorts (hereafter ‘populations’) displayed minimal strain-sharing rates (median 0%) (Kruskal–Wallis test, *n* = 26,218, *χ*^2^ = 11,420, *P* < 2.2 × 10^−16^, post hoc Dunn tests, adjusted P value (*P*_adj_) < 0.05; Fig. 1f and Supplementary Table 7). This highly significant pattern is confirmed by the percentage of individuals not sharing a single detectable strain: whereas only 4% of mother–offspring pairs had no detected strain-sharing event, no strains were shared by 82% of pairs with no obvious person-to-person contact in the same population, and by up to 97% of individuals in different populations (Fig. 1f). Person-to-person strain sharing thus follows a social distance-based gradient across shared environments and kinship that is notably stronger than that observed by species-level microorganism divergence (beta diversity indices, Kruskal–Wallis tests with post hoc Dunn tests, *P*_adj_ < 0.05; Extended Data Fig. 4b and Supplementary Table 8). Overall, our integrated analysis highlights the relevance of direct person-to-person interaction and social-interaction networks in shaping the gut microbiome of single individuals.

## Extensive mother–offspring transmission

**Fig. 2:**
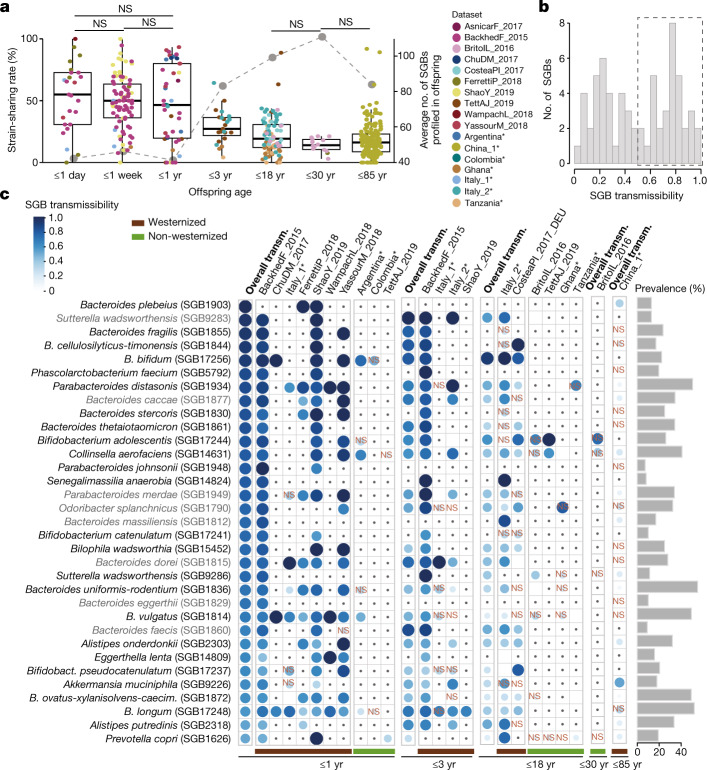
Mother–offspring sharing of the gut microbiome. **a**, Mother–offspring strain-sharing rates (left axis; box plots and non-grey dots) decrease, whereas species richness (right axis; grey dots) in offspring increases, as a function of offspring age (17 datasets in 14 countries). The median number of SGBs profiled by StrainPhlAn in the offspring is used as a proxy for richness (right axis). Kruskal–Wallis test, *n* = 448, *χ*^2^ = 156, *P* < 2.2 × 10^−16^, post hoc Dunn tests; NS, not significant (*P*_adj_ ≥ 0.05); all other comparisons are significant (Supplementary Table 10). In box plots, box edges delineate lower and upper quartiles, the centre line represents the median and whiskers extend to 1.5 times the IQR. Novel datasets from the present study are highlighted with asterisks. **b**, The distribution of mother–infant SGB transmissibility in the first year of life. **c**, A panel of 33 SGBs that are highly maternally transmitted during their first year of life (SGB transmissibility >0.5 and significantly higher mother–infant transmissibility than unrelated mother–infant transmissibility; Methods) showing their transmissibility (transm.) in specific datasets (separated by westernized-lifestyle status) and in other age categories. NS, non-significant SGB transmissibility in the category (*χ*^2^ test on the number of transmitted and non-transmitted SGBs between mother–offspring pairs and between unrelated mother and offspring pairs, Supplementary Table 16). Only comparisons with at least three possible transmissions (species shared by at least three mother–offspring pairs) are shown; comparisons with less than three possible transmissions are marked with a dot. Prevalence is defined as the percentage of mother–offspring samples in which the SGB was detected. Novel datasets from the present study are highlighted with asterisks. SGB names in grey use a strain identity threshold of 5% inter-individual nGD (Supplementary Table 4). *B. cellulosilyticus-timonensis*, *Bacteroides cellulosilyticus* and *Bacteroides timonensis*; *Bacteroides uniformis-rodentium*, *Bacteroides uniformis* and *Bacteroides rodentium*; *B. pseudocatenulatum*, *Bifidobacterium pseudocatenulatum*; *B. ovatus-xylanisolvens-caecim.*, *Bacteroides caecimuris*.

Mother-to-offspring microbiome transmission has been described^9–11,29,32,41^, and our expanded sample set (3,598 samples from 711 mother–offspring pairs, including 636 novel stool samples; Fig. 1a) enabled further generalization of the previously reported patterns. We found a remarkable negative correlation between the strain-sharing rate and the age of the offspring (Spearman’s test, *n* = 448, *ρ* = −0.52, *P* < 2.2 × 10^−16^; Kruskal–Wallis test, *χ*^2^ = 156, *P* < 2.2 × 10^−16^; Fig. 2a) despite the increase on the number of mother–offspring shared species with offspring age (median = 17 shared species in the first year of life, 37 up to 3 years of age, and 57 up to 18 years of age), suggesting the accumulation of species putatively originating from other sources by the offspring. During the first year of life, infants shared with their mothers half of the strains of the species found in both the infant and the mother microbiomes (strain-sharing rate) and 16% of the strains detected in the infants putatively originated from the mother (Extended Data Fig. 6a and Supplementary Table 10), with only slight non-significant reductions in strain-sharing rates after the first few days^9,12^ (65%, 50% and 47% median strain-sharing rates at 1 day, 1 week, and 1 year, respectively; post hoc Dunn tests, *P*_adj_ ≥ 0.05, Supplementary Table 10). In concordance with the reduced post-weaning physical intimacy and the infant’s expanding motor activities^42^, strain sharing then decreased to 27% at 1–3 years of age (Fig. 2a). Mother–offspring strain-sharing rates stabilized after 3 years of age (19% for up to 18 years of age and 14% for up to 30 years of age; Fig. 2a), approaching those observed between household members (12%; Fig. 1f). Whereas ample strain sharing at birth confirms the substantial extent of maternal microbiome seeding of the infant’s gut, strain sharing remained significant in senior individuals (50–85 years of age), with non-cohabiting mother–offspring pairs still sharing significantly more strains than with unrelated mothers (16% versus 8%; Wilcoxon rank-sum test, *n* = 17,177, *r* = 0.09, *P* = 4.1 × 10^−35^; Extended Data Fig. 6b). This may be the result of the combined effect of long-lasting maternal microorganism imprinting at birth and strain transmission driven by shared social environments later in life.

Potential effectors of maternal gut microbiome transmission include lifestyle and mode of delivery^14,29^. Although the newly sequenced non-westernized populations reinforced the well-documented westernization-associated reduction in microorganism diversity^43–45^ both in mothers (Wilcoxon rank-sum test, *n* = 721, *r* = −0.37, *P* = 7.4 × 10^−24^) and their offspring (*P*_adj_ < 0.05, Extended Data Fig. 6c and Supplementary Table 11), we noticed no differential mother–offspring strain-sharing rates in most age categories (Wilcoxon rank-sum tests, *P*_adj_ ≥ 0.05 for all age categories except for 3–18 years of age; Supplementary Table 12). Indeed, similar numbers of strains were maternally transmitted in westernized and non-westernized communities (Wilcoxon rank-sum tests, *P*_adj_ ≥ 0.05 for all age categories except for 3–18 years; Supplementary Table 13). The high microbiome diversity in non-westernized populations thus does not seem to be maintained by maternal transmission of microbiome strains but might be gained by closer interaction with more individuals. By contrast, we did confirm an association between mode of delivery and mother–offspring strain sharing early in life: vaginally delivered infants (up to 1 year of age) displayed significantly higher strain-sharing rates with their mothers (Wilcoxon rank-sum tests, *P*_adj_ < 0.05; Extended Data Fig. 6d and Supplementary Table 14). However, paralleling the age-associated decreased influence of mode of delivery on the infants’ microbiome^46^, no difference was detected after 3 years of age (*n* = 56, *r* = 0.2, *P*_adj_ = 0.18; Supplementary Table 14). Therefore, whereas vaginal delivery provides evident gut microbiome imprinting via maternal transmission early in life, lifestyle differences—including divergent hygiene and built-environment sanitation levels—do not substantially affect microbiome transmission rates.

Transmission from mothers to offspring (defined on offspring of up to 1 year of age—before the reduction in strain sharing; Fig. 2a) varied largely among species (Fig. 2b), but SGB transmissibility was rather consistent across datasets (pairwise Spearman’s tests, *ρ* = 0.59–0.83, *P*_adj_ < 0.05; Supplementary Table 15), revealing species transmissibility as a specific trait of microorganisms. All highly transmitted SGBs (51% SGBs, transmissibility greater than 0.5 and significantly higher mother–infant transmissibility than unrelated mother–infant transmissibility; Methods) across 10 datasets belonged to characterized species (kSGBs) (Chi-squared tests, *n* = 33, *P*_adj_ < 0.05; Fig. 2c and Supplementary Table 16), mostly of the genera *Bacteroides* and *Bifidobacterium* (*n* = 16 (48%) and *n* = 5 (15%) SGBs, respectively; Fig. 2c). As a case in point, *Bacteroides vulgatus* (SGB1814) and *Bifidobacterium longum* (SGB17248) were detected in all westernized datasets as significantly transmitted between mothers and infants (Chi-squared tests, *P*_adj_ < 0.05; not prevalent enough in non-westernized datasets to assess transmissibility; Fig. 2c, Supplementary Table 16 and Methods). By contrast, other SGBs detected in infants—such as *Roseburia intestinalis* (SGB4951), which was found in 13 children and 102 mothers—were extremely rarely maternally transmitted (Supplementary Table 9). The highly maternally transmitted SGBs were found to be gradually less shared between mothers and older offspring (Fig. 2c and Supplementary Table 16), but significant transmissibility of 52% of the highly maternally transmitted SGBs was detected even in senior individuals (50–85 years old) not cohabiting with their mothers (Fig. 2c and Supplementary Table 16).

## Cohabitation drives transmission

**Fig. 3:**
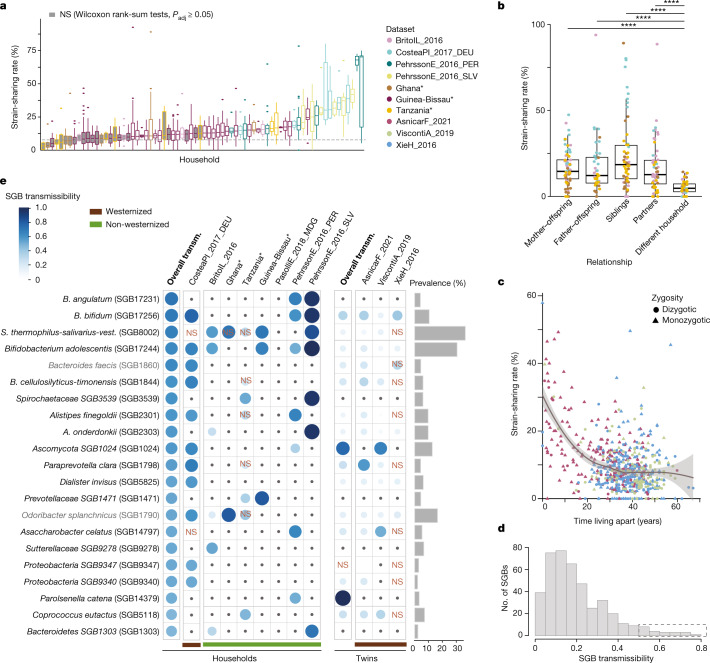
Within-household and between-household gut microbiome transmission. **a**, Pairwise person-to-person strain-sharing rates (number of shared strains/number of SGBs in common × 100%) in 72 households with at least four cohabiting individuals (*n* = 883). The dashed line shows the median sharing rate among individuals in different households of the same village. Grey-filled boxes represent households with intra-household strain-sharing rates that are not significantly higher than inter-household sharing rates in the same population (Wilcoxon rank-sum two-sided tests, *P*_adj_ ≥ 0.05; Supplementary Table 17). In box plots, box edges delineate lower and upper quartiles, the centre line represents the median and whiskers extend to 1.5 times the IQR. Novel datasets from the present study are highlighted with asterisks. **b**, Strain-sharing rates between individuals in households. Post hoc Dunn two-sided tests, *n* = 282, ****P_adj_ < 10^−4^ (Supplementary Table 18). In box plots, box edges delineate lower and upper quartiles, the centre line represents the median and whiskers extend to 1.5 times the IQR. **c**, Strain-sharing rate in non-cohabiting adult twins (*n* = 1,734) decreases as a function of the time spent living apart (loess curve). The shaded area shows the 95% confidence interval. **d**, Histogram of household SGB transmissibility. **e**, A panel of 21 SGBs that are highly transmitted in households (SGB transmissibility >0.5 and significantly higher intra-household than inter-household transmissibility) showing their transmissibility in specific datasets and in non-cohabiting adult twins. NS, non-significant SGB transmissibility in the category (Chi-squared test on the number of transmitted and non-transmitted SGBs between household pairs and between pairs in different households; Supplementary Table 20). Only comparisons with at least three possible transmissions (species shared by at least three cohabiting pairs) are shown; comparisons with less than three possible transmissions are marked with a dot. Prevalence is defined as the percentage of samples in which the SGB was detected. Novel datasets from the present study are highlighted with asterisks. SGB names in grey use a strain identity threshold of 5% inter-individual nGD (Supplementary Table 4). *S. thermophilus-salivarius-vest.*, *S. thermophilus*, *S. salivarius* and *S. vestibulari*.

Gut microbiome similarities among household members are well documented^45,47–49^, but because of the missing strain-level resolution, most studies have not been able to conclude whether similarities at higher taxonomic levels reflected microorganism transmission or rather modulation by similar conditions (for example, genetics or diet). To examine horizontal gut microbiome transmission, we assessed strain sharing among 883 cohabiting individuals (up to 4 years old) in 212 households from 8 populations on 4 continents (Fig. 1a) with remarkably diverse lifestyles: from traditional subsistence in rural areas^17,23,30,34^, to crowding conditions in large developing cities^23^ and medium-sized industrialized affluent cities^27^. The majority of households displayed significantly higher person-to-person strain-sharing rates (between 11% and 71%) among cohabiting members than with non-cohabiting individuals of the same population (64% households, Wilcoxon rank-sum tests, *P*_adj_ < 0.05; 28% to 778% median increase in strain-sharing rates compared with among different households; Fig. 3a and Supplementary Table 17). Weaker differences were found for species-level microbiome similarities (beta diversity indices; Extended Data Fig. 4b) between individuals sharing households and non-cohabiting individuals (3% to 9% increase, Kruskal–Wallis tests with post hoc Dunn tests, *P*_adj_ < 0.05; Supplementary Table 8). Although person-to-person strain sharing varied largely across households (Kruskal–Wallis test, *n* = 1,632, *χ*^2^ = 223, *P* = 2.8 × 10^−45^), this was only slightly associated with westernized lifestyles (Wilcoxon rank-sum test, *n* = 1,632, *r* = −0.22, *P* = 2.2 × 10^−18^), possibly pointing to limited effects of environmental and social variables. Strain sharing between cohabiting individuals decreased with age (Wilcoxon rank-sum test for under 4 years of age versus 4 years and older, *n* = 1,843, *r* = −0.12, *P* = 1.3 × 10^−7^), supporting a lower colonization resistance in early life^6,32^. By contrast, the number of strains of non-family origin (defined as those not shared with any household member) increased with age, as expected with increased cumulative exposure (Wilcoxon rank-sum test for under 4 years of age versus 4 years and older, *r* = 0.20, *P* = 4.9 × 10^−8^).

We next assessed strain sharing between parents and offspring, between siblings and between partners in the four populations in which kinship was known. All family relationships displayed significantly higher strain-sharing rates than different-household comparisons (post hoc Dunn tests, *n* = 282, *P*_adj_ < 0.05; Fig. 3b and Supplementary Table 18), but no significant differences were detected among them. Maternal and paternal strain-sharing rates were similar in children 4 years of age and older, and there was slightly (but not significantly) higher strain sharing between younger (that is, less richly colonized), genetically related siblings than between partners. To assess the extent to which co-housing impacts strain sharing later in life, we analysed metagenomes from non-cohabiting adult twins who had lived together in the past (1,734 samples from three published cross-sectional datasets^2,25,33^ in the United Kingdom), including both monozygotic and dizygotic twins. Strain sharing between twin pairs decreased significantly with the number of years spent living apart (Spearman’s test, *n* = 708, *ρ* = −0.30, *P* = 9.2 × 10^−15^) and after accounting for their age (generalized linear model (GLM), *n* = 648, *β* = −0.58, *P* = 7.1 × 10^−18^; Fig. 3c). There was a moderate genetic effect beyond the influence of past cohabitation, with monozygotic twins displaying higher strain-sharing rates decades after cohabitation than dizygotic twins (Wilcoxon rank-sum tests, *P*_adj_ < 0.05; Extended Data Fig. 7 and Supplementary Table 19). Finally, the more gradual decline in age-associated strain sharing when partialling out the number of years twins have lived apart (GLM, *n* = 648, *β* = −3.9 × 10^−3^, *P* = 0.02) provides further evidence for the effect of cohabitation on microbiome transmission in adults and its larger quantitative effect than genetics and age. Strain sharing among adult twins might therefore be more the result of past cohabitation than of a long-lasting effect of shared transmission from their parents.

A panel of 21 SGBs (4% of assessed SGBs) from 10 different bacterial genera were highly transmitted between household members (SGB transmissibility >0.5 and significantly higher intra-household than inter-household transmissibility; Fig. 3d,e, Supplementary Table 20 and Methods). Household SGB transmissibility was not consistent across datasets (pairwise Spearman’s tests, *P*_adj_ ≥ 0.05; Supplementary Table 21), in contrast to mother-to-infant transmissibility, and we observed large differences in SGB transmissibility between westernized and non-westernized lifestyles (Fig. 3e) in concordance with their divergent microbiome composition^30,45,50,51^. A high portion (38%) of highly transmitted SGBs were species without characterized isolates or genomes (uSGBs) for the species (*n* = 1) or genus (*n* = 7) they belong to. Most highly transmitted *Bifidobacterium* and *Bacteroides* species in households coincided with those found highly transmitted from mother to offspring (Figs. 2c and 3e), suggesting these are efficient spreaders regardless of transmission mode, in contrast to *Bifidobacterium angulatum* (SGB17231), which emerged as preferentially transmitted across households. Notably, SGBs that were highly transmitted within households tended to remain shared among twin pairs who moved apart (94% of the 21 highly transmissible SGBs; Fig. 3e and Supplementary Table 20), supporting the partial persistence of transmitted strains.

## Microorganism transmission along populations

**Fig. 4:**
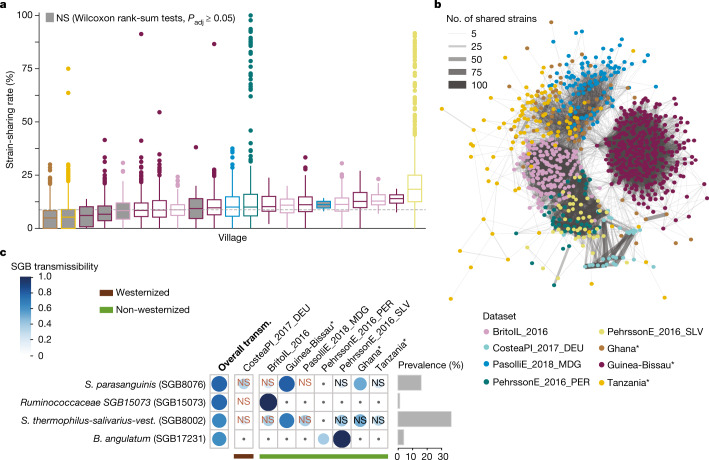
Gut microbiome transmission along villages and populations. **a**, Person-to-person strain-sharing rates in different households of a village (*n* = 1,132). The dashed line shows the median strain-sharing rate among individuals in different villages of the same dataset. In box plots, box edges delineate lower and upper quartiles, the centre line represents the median and whiskers extend to 1.5 times the IQR. Grey-filled boxes show non-significant differences between the within village and inter-village person-to-person strain-sharing rate (Wilcoxon rank-sum two-sided tests, *P*_adj_ ≥ 0.05; Supplementary Table 23). **b**, Gut microbiome strain-sharing unsupervised network of individuals in household datasets displaying population structure. Line width is proportional to the number of shared strains. **c**, Highly transmitted SGBs between individuals in different households (SGB transmissibility >0.5 and significantly higher intra-population than inter-population transmissibility), and transmissibility of these SGBs in specific datasets (classified by westernization status). NS, non-significant SGB transmissibility in the category (Chi-squared two-sided tests on the number of transmitted and non-transmitted SGBs between inter-household pairs and between pairs in different datasets; Supplementary Table 24). Only comparisons with at least three possible transmissions (species shared by at least three pairs) are shown; comparisons with less than three possible transmissions appear with a dot. Prevalence is defined as the percentage of samples in which the SGB was detected. Novel datasets from the present study are highlighted with asterisks.

Non-cohabiting individuals in a village displayed non-negligible strain sharing of gut microbiome, in contrast to individuals with no presumed shared environments, albeit at notably lower rates than same-household members (Kruskal–Wallis test, *n* = 1,132 samples across 7 datasets, *χ*^2^ = 1,721, *P* < 2.2 × 10^−16^; post hoc Dunn tests, *P*_adj_ < 0.05; Extended Data Fig. 8a and Supplementary Table 22). Whereas intra-village strain-sharing rates were largely variable within populations (Fig. 4a), in 67% of villages, individuals from different households in the same villages had significantly higher strain-sharing rates than those in different villages (Wilcoxon rank-sum tests, *P*_adj_ < 0.05; Supplementary Table 23) in 5 out of the 7 populations assessed. Person-to-person microbiome transmission thus also occurs upon interaction between more distant contacts, and is potentially affected by population structures^4,17^. Indeed, we found that microbiome strain transmission within and between populations recapitulated host population structures (PERMANOVA on Euclidean distance in unsupervised strain-sharing network, *n* = 951, *R*^2^ = 46%, *P* = 10^−2^; Fig. 4b and Methods) at a markedly stronger degree than that of species sharing (PERMANOVA on Euclidean distance on species sharing network, *n* = 951, *R*^2^ = 11%, *P* = 10^−2^; Extended Data Fig. 8b).

Although only 4 SGBs (0.8%) displayed high intra-population transmissibility overall (SGB transmissibility >0.5 and significantly higher intra- than inter-population transmissibility; Fig. 4c, Supplementary Table 24 and Methods), intra-population species transmissibility was highly consistent across datasets (pairwise Spearman’s tests on SGB intra-population transmissibility by dataset, *ρ* > 0, *P*_adj_ < 0.05; Supplementary Table 25). Three highly transmitted SGBs are known members of the human microbiome: *B. angulatum* (SGB17231, 4% prevalence), *Streptococcus parasanguinis* (SGB8076, a species with opportunistic pathogen representatives^52^, 16%), and *S. thermophilus*, *S. salivarius* and *S. vestibularis* (SGB8002, including some strains commonly used as probiotic^53^, 37%), suggesting that both health-associated and potential pathogenic species can be efficient spreaders. A so-far uncharacterized species of the Ruminococcaceae family was also among the highly transmitted SGBs (SGB15073, 1% prevalence). Although *S. thermophilus*, *S. salivarius*, *S. vestibularis* and *B. angulatum* also appeared as highly transmitted in households, the specific high transmissibility of *S. parasanguinis* and SGB15073 among non-cohabiting individuals (Figs. 2c and 3e) suggests distinct spreading mechanisms.

## Mostly horizontal oral transmission

**Fig. 5:**
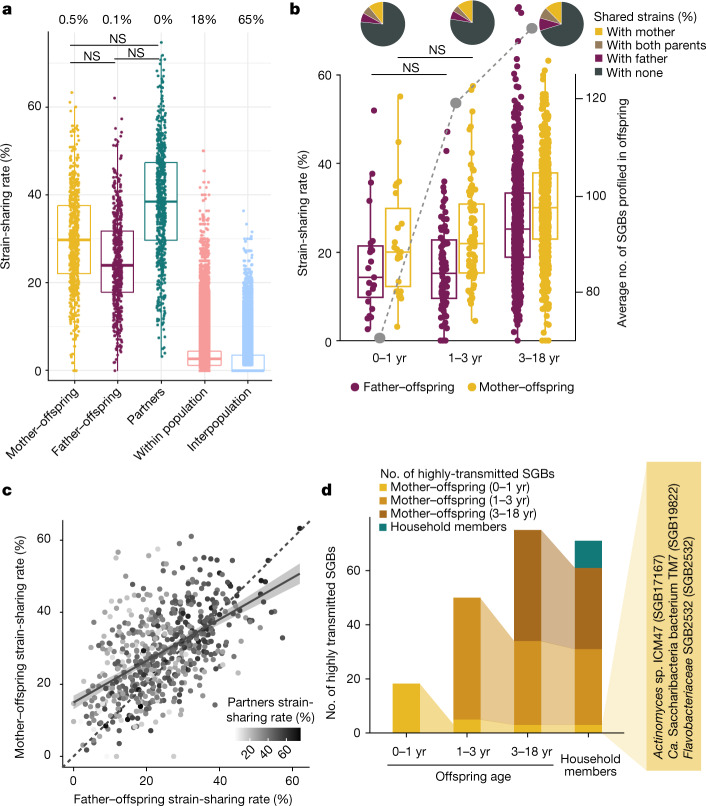
Transmission of the oral microbiome. **a**, Person-to-person strain-sharing rates (number of shared strains/number of SGBs in common × 100%) across relationships (*n* = 2,069). All comparisons are statistically significant unless stated otherwise (Kruskal–Wallis test, *n* = 26,218, *χ*^2^ = 11,420, *P* < 2.2 × 10^−16^, post hoc Dunn two-sided tests, *P*_adj_ < 0.05; Supplementary Table 28). Numbers along the top show the percentage of pairs between which no strain-sharing event was detected. **b**, Mother–offspring and father–offspring sharing rates (number of shared strains/number of SGBs in common × 100%) (*n* = 2,069) (left axis; box plot and non-grey dots) and median number of SGBs detected in the offspring (right axis; grey dots). Post hoc Dunn two-sided tests, Supplementary Table 29. All comparisons are statistically significant after correction for multiple testing unless stated otherwise. In box plots, box edges delineate lower and upper quartiles, the centre line represents the median and whiskers extend to 1.5 times the IQR. Pie charts show the percentage of strains shared between pairs of individuals. **c**, Strain sharing across cohabiting individual relationships are positively correlated (Spearman’s two-sided tests, mother–offspring and father–offspring: *n* = 637, *ρ* = 0.52, *P* < 2.2 × 10^−16^; mother–offspring and partners: *n* = 611, *ρ* = 0.21, *P* = 1.2 × 10^−7^; father–offspring and partners: *n* = 611, *ρ* = 0.38, *P* < 2.2 × 10^−16^). Dashed line is the diagonal, where mother–offspring strain-sharing rate is equal to father–offspring strain-sharing rate. The shaded area shows the 95% confidence interval. **d**, The persistence of highly transmitted SGBs (SGB transmissibility >0.5 and significantly higher intra-household than inter-household transmissibility) between mother and offspring across age categories and among household members who are at least four years of age. *Ca.*, *Candidatus*.

**Extended Data Fig. 5:**
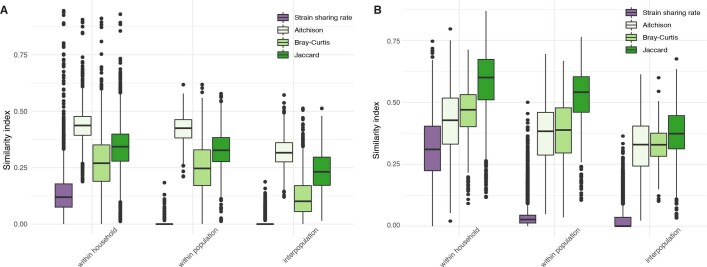
Strain and species-level similarity across relationships. **A**) Gut microbiome strain sharing rates and species-level similarity metrics (Aitchison similarity, Bray-Curtis similarity, and Jaccard binary similarity) between individuals in the same household (“within household”) as compared to unrelated non-cohabiting individuals in different villages of the same population (“within population”) and individuals in different populations (“interpopulation”). For comparability with strain sharing rates, species-level comparisons are depicted as similarity indices (1 - distance or dissimilarity). All comparisons are significant (Padj<0.05, Kruskal-Wallis tests with Post-hoc Dunn tests, Table S8). The social-distance based gradient followed by strain sharing rates is notably stronger than that observed by species-level similarity metrics (Table S8). Boxes: lower and upper quartiles, middle line: median; whiskers: 1.5 × IQR. **B**) Oral microbiome strain sharing rates and species-level similarity metrics (Aitchison, Bray-Curtis, and Jaccard binary similarities) between individuals in the same household (“within household”) as compared to unrelated non-cohabiting individuals in different villages of the same population (“within population”) and individuals in different populations (“interpopulation”). For comparability with strain sharing rates, species-level comparisons are depicted as similarity indices (1 - distance or dissimilarity). All comparisons are significant (Padj<0.05, Kruskal-Wallis tests with Post-hoc Dunn tests, Table S28). Boxes: lower and upper quartiles, middle line: median; whiskers: 1.5 × IQR.

Oral microbiome strains are probably more easily transmitted among individuals than gut strains, as saliva can be a direct vehicle^54^, but person-to-person oral microbiome transmission remains underexplored^17,54,55^. We assessed the patterns of oral strain sharing in 1,929 newly sequenced metagenomes from households in the United States (USA dataset) together with 140 saliva metagenomes publicly available from a population in the Fiji islands^17^ by strain-level profiling of 252 SGBs (Methods). We detected a strain-sharing rate gradient across shared environments and kinship, similar to that observed for gut microbiome strain sharing: cohabiting individuals displayed 32% median oral strain-sharing rates, whereas non-cohabiting individuals in the same or different populations shared 3% and 0%, respectively (Kruskal–Wallis test, N = 2,069, *χ*^2^ = 41,317, P < 2.2 × 10^−16^; Fig. 5a). Cohabiting individuals thus feature 10 times higher oral strain-sharing rates than non-cohabiting individuals in the same population, in contrast to less than 0.5 times higher species-level microbiome similarity (Extended Data Fig. 5b and Supplementary Table 26), suggesting that strain transmission between household members is a stronger driver of genetic microbiome composition than species-level microbiome convergence through similar conditions and lifestyles. In addition, less than 0.5% of same-household members did not share a single strain, in contrast to 18% of intra-population pairs and 65% of inter-population pairs; this indicates that person-to-person transmission of bacterial oral strains occurs more frequently than gut microbiome transmission (Fig. 1f).

Distinct age- and kinship-associated patterns emerged: in contrast to the gut microbiome pattern, oral strain-sharing rates increased with offspring age (Spearman’s test, *n* = 658, *ρ* = 0.15, *P* = 1.9 × 10^−4^ for mother–offspring and *n* = 643, *ρ* = 0.24, *P* = 7.1 × 10^−10^ for father–offspring), especially after 3 years of age (Kruskal–Wallis test, *χ*^2^ = 31, *P* = 1.7 × 10^−7^ for mother–offspring, *χ*^2^ = 58, *P* = 2.4 × 10^−13^ for father–offspring, post hoc Dunn tests, Supplementary Table 27), coinciding with the increasing accumulation of microorganism species in the offspring’s oral microbiome (from a median of 49 shared species between mothers and offspring and 55 shared species between fathers and offspring up to 1 year of age, to a median of 85 shared species between mothers and offspring and 86 shared species between fathers and offspring up to 18 years of age; Spearman’s test, *n* = 658, *ρ* = 0.21, *P* = 6.2 × 10^−8^; Fig. 5b). No significant differences were detected among different types of relationships (post hoc Dunn tests, *P*_adj_ ≥ 0.05; Supplementary Table 28), but strain-sharing rates were slightly higher between partners (median 38%) than for the younger offspring with their mothers (30%) and fathers (24%; Fig. 5a) probably reflecting greater intimacy^54^. Mother–offspring species sharing rates tended to be higher than father–offspring species sharing rates across age ranges (post hoc Dunn tests, *P*_adj_ < 0.05; Supplementary Table 29), potentially as a result of closer contacts and imprinting through breastfeeding. However, although the proportion of strains shared with both partners increased slightly with offspring age (6% below 1 year to 8% below 18; Fig. 5b), even more strains were shared with each parent separately (17–21% with mothers and 13–17% with fathers). Overall, parental strain transmission does not seem to particularly seed oral microbiome assembly in early life, but rather appears to exploit horizontal transmission modes that are also dependent on the duration of the contact.

Intra-family oral strain transmission varied largely across households (0–75%), and although conclusions on lifestyle associations cannot be drawn on the basis of the two datasets available with disparate sample sizes, we did find significant correlations between strain sharing in households across all types of kinship assessed (Fig. 5c). Mother–offspring strain-sharing rates correlated with father–offspring strain-sharing rates (Spearman’s test, *n* = 637, *ρ* = 0.52, *P* < 2.2 × 10^−16^) and with partner strain-sharing rates (Spearman’s test, *n* = 611, *ρ* = 0.21, *P* = 1.2 × 10^−7^). Also, father–offspring strain-sharing rates correlated with those between partners (Spearman’s test, *n* = 611, *ρ* = 0.38, *P* < 2.2 × 10^−16^). Closely interacting households thus seem to favour oral strain transmission among all cohabiting individuals regardless of kinship.

We next assessed parent-to-offspring and household oral species transmissibility (Supplementary Table 30). Eighteen SGBs (half of which were uSGBs) from 16 different genera were significantly highly shared between mothers and their infants up to 1 year of age (19% of total SGBs assessed, SGB transmissibility>0.5 and significantly higher intra-mother–offspring pair than inter-mother–offspring pair transmissibility; Fig. 5d), including two *Prevotella* species (*Prevotella histicola* (SGB1543) and *Prevotella pallens* (SGB1564)) and two largely uncharacterized *Actinomyces species* (SGB17132 and SGB17167; Supplementary Table 31). Although SGB transmissibility up to 1 year of age showed a strong correlation with that at 1–3 years of age (Spearman’s test, *n* = 95, *ρ* = 0.73, *P* < 2.2 × 10^−16^) and between 3 and 18 years of age (*n* = 95, *ρ* = 0.78, *P* < 2.2 × 10^−16^), only five species persisted as highly transmitted between the first (up to 1 year) and second (1 to three years) age bin and three persisted to the third (3 to 18 years) age bin, with up to 68 further species appearing (Fig. 5d and Supplementary Table 31). These 68 later-emerging species were highly concordant with the 70 species (including 28 uSGBs) displaying significantly high household transmissibility (28% of total SGBs assessed; Supplementary Table 32), including the three persisting highly maternally transmitted SGBs. By contrast, no species was highly transmitted among non-cohabiting individuals (Supplementary Table 30). Overall, three under-characterized SGBs thus exhibited consistently strong oral transmission potential: *Actinomyces* sp. ICM47 (SGB17167), *Candidatus* Saccharibacteria bacterium TM7 (SGB19822), and a uSGB of the family Flavobacteriaceae (SGB2532) (Fig. 5d and Extended Data Fig. 9).

## Phenotypes linked to transmission modes

The transmissibility of gut species was highly consistent across geographically distant datasets with diverse lifestyles (Spearman’s tests, *P*_adj_ < 0.05; mother-to-infant: 71%, intra-population: 75% significant associations; Supplementary Tables 15, 21 and 25, with transmissibility estimates ranging between 0 and 100%). At the same time, gut species were often preferentially transmitted through specific modes^56^ (23% SGBs were highly transmitted through more than 1 mode; Figs. 2c, 3e and 4c). By contrast, highly transmitted oral SGBs across transmission modes were largely overlapping (Fig. 5d). Species transmissibility did not seem to predominantly follow a mass-action model of transmission—neither median relative abundance nor the prevalence of a species in populations was positively associated with its transmissibility (Spearman’s one-sided tests, *P*_adj_ ≥ 0.05; Supplementary Table 33).

**Fig. 6:**
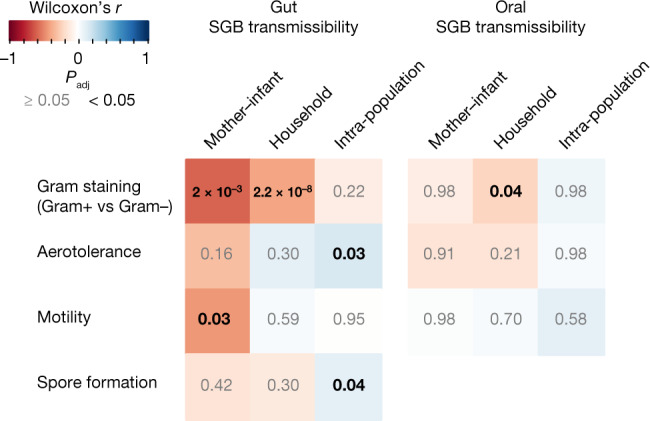
Association between gut and oral species transmissibility and phenotypical properties. SGB phenotypes were inferred using Traitar^60^ (Methods). Association between SGB transmissibility and predicted phenotypes was assessed with Wilcoxon rank-sum two-sided tests on the 25% of SGBs displaying the highest transmissibility and compared with the 25% of SGBs displaying the lowest transmissibility for each transmission mode and environment. Colours represent the Wilcoxon *r* statistics; significant *P*_adj_ values are shown in black (*P*_adj_ < 0.05) and in grey otherwise.

The absence of a direct link between prevalence and transmissibility is consistent with species transmissibility through different modes being a specific trait, so we next explored whether phenotypic properties associated with persistence in the environment^3,4^ could better account for the patterns we detected. As 58% of the gut and 24% of the oral SGBs that we profiled at the strain level have not yet been cultured, we inferred bacterial phenotypes on the basis of their genome sequences (Methods). The predicted phenotypes showed more than 90% concordance with experimentally determined traits in cases where those were available (Supplementary Table 34 and Methods). Gut and oral microbiome transmission modes were associated with specific phenotypic properties (Fig. 6). Gram-negative bacteria—generally more resistant to sanitizers and disinfectants^57^—displayed enhanced gut maternal and household transmissibility (Wilcoxon rank-sum tests on first versus fourth quartiles of SGB transmissibility, *n* = 35, *r* = −0.59, *P*_adj_ = 2.0 × 10^−3^ and *n* = 213, *r* = −0.40, *P*_adj_ = 2.2 × 10^−8^, respectively), together with increased oral household transmissibility (*n* = 126, *r* = −0.22, *P*_adj_ = 0.04). Longer-range gut intra-population transmissibility required more powerful environmental survival mechanisms—that is, aerotolerance and spore formation (*n* = 268, *r* = 0.16, *P*_adj_ = 0.03 and *n* = 280, *r* = 0.10, *P*_adj_ = 0.04, respectively). With less than 10% of profiled gut SGBs being predicted as oxygen-resistant in contrast to more than 66% of oral ones, aerotolerance was not associated with transmissibility of oral SGBs (Fig. 6). Finally, the motile species that are frequent but unstable inhabitants of the infant gut^58^ were less frequently transmitted from mothers to offspring than non-motile SGBs (*n* = 35, *r* = −0.43, *P*_adj_ = 0.03), which could be beneficial given the link between motility and virulence^59^. Overall, our results suggest that microorganism phenotypic properties promoting survival in the environment at least partially modulate person-to-person gut microbiome transmission dynamics, whereas a notably weaker link was found for oral microbiome transmission.

## Conclusion

Our integrative multi-cohort study of microbiome transmission across diverse populations shows extensive previously overlooked person-to-person transmission. This corroborates already suggested hypotheses^3–5,16^ and reveals that the transfer of microorganism strains among individuals in long-lasting close contact is a major driver in shaping the personal genetic makeup of the microbiome, and thus of the corresponding metabolic and host–microorganism interaction potential. Although strain sharing was, as expected, greatest between mother and infant gut microbiomes during the first year of life^9,10,12,29,32^ (median of 50%), shared strains also accounted for 12% and 32% of the gut and oral microbiome species in common between cohabiting individuals, respectively (Figs. 1f and 5a). Such an effect might be induced by close physical interaction even when such interaction started only in adulthood (13% and 38% gut and oral strain sharing between partners respectively; Figs. 3b and 5a) and is partially reversible over long periods, with twins decreasing their initial strain sharing of around 30% to about 10% over 30 years of living apart (Fig. 3c). Because unrelated individuals in different populations or even in different villages of the same population share hardly any strains (0% median strain-sharing rate), our results highlight a non-negligible effect of social interactions in shaping the microbiome, which could have a role in microbiome-associated diseases, and warrants consideration of person-to-person strain transmission in human microbiome studies.

By contrast, we found little influence of divergent lifestyles on microbiome transmission dynamics: despite massive microbiome composition differences in populations loosely defined as westernized or non-westernized^34,43,51^ on the basis of characteristics such as diet, access to medical facilities and drugs, and hygiene conditions (Methods), we found remarkably similar vertical and horizontal strain-sharing rates. Larger, diverse cohorts and more detailed metadata on participants’ lifestyles and cultural practices are needed to ensure the robustness of this finding, but our results might point to similar microorganism colonization resistance in different populations that could be of greater importance in establishing durable colonization than the intrinsic rates of transmission events. Our results also suggest that the higher richness of microorganisms observed in non-westernized communities^34,43^ is not promoted by enhanced transmission from other household members, but is rather a consequence of the interaction with the environment as well as diets and lifestyles supporting microorganism diversity.

Species showing particularly high transmissibility (Figs. 2c, 3e, 4c and 5d) should be the starting point for a deeper understanding of the genomic and phenotypic characteristics that can in turn inform transmission mechanisms. Although our study could not resolve whether person-to-person microbiome transmission was direct or its directionality, it provided a systematic overview of microbiome transmission in humans. Further insight into person-to-person microbiome transmission and its directionality could be obtained using specific study designs modelling changes in routine social-interaction networks in humans (for example, following household changes) or in other social animals. The improved strain tracking methods we used that included strain-level profiling of so-far uncultured species^39^ and species-specific definitions of strain based on phylogenetic distances enabled us to scale to large numbers of samples corresponding to more than 800,000 strains. Nonetheless, future studies with whole-genome resolution enabled by deeper sequencing, long-read technologies or single-cell approaches may enable further clarification and refinement of these findings. Overall, our results reinforce the hypothesis that several diseases and conditions that are currently considered non-communicable should be re-evaluated^5^, and that accounting for transmissibility and social network structure will improve the design of future microbiome investigations and modulation approaches.

## Methods

### Metagenomic datasets

A total of 9,715 samples from 31 human metagenomic datasets (total: 5.17 × 10^11^ reads, average: 5.32 × 10^7^ reads per sample) with available metadata to enable assessment of microbiome transmission between healthy mothers and offspring, households, twin pairs, villages and populations (that is, cohabitation information) were selected for inclusion in this study (Supplementary Tables 1 and 2). We also included publicly available stool shotgun metagenomic datasets with samples from at least 15 healthy individuals to whom no intervention (such as antibiotic or drug treatment, or specific diet) was performed, with at least 2 of the samples taken less than 6 months apart to assess within-subject strain retention and set species-specific operational definitions of strain identity 25 datasets were publicly available, three of which were expanded in this study with 14 (FerrettiP_2018^9^), 32 (Ghana dataset^34^) and 61 (Tanzania dataset^34^) samples. Newly included samples were collected and processed following the protocols described in the original publications. In addition, eight datasets (total: 2,800 samples) were newly collected and sequenced in the context of this study as described below, using similar methods (although differences in sample processing, DNA extraction and sequencing library preparation do not directly affect the phylogenetic distances that we use to infer strain sharing).

#### Consistent metadata collection and organization

We retrieved the metadata on sample and subject identifiers, time points, participant’s age, gender, mode of delivery (vaginal or caesarian section), family identifiers, family relationships, twin zygosity and age at which twins moved apart, village, and country from curatedMetagenomicData 3.0.0 (ref. ^61^) when included in the resource, and from the publications’ supplementary materials or specified repository otherwise. Metadata of all metagenomes, including newly sequenced samples, were curated and organized in the curatedMetagenomicData format and are available in Supplementary Table 2. Partners were defined as couples that share a household. Populations were classified on the basis of their westernization status (westernized or non-westernized), considered as the adoption of a westernized lifestyle and not in geographical terms, and defined as intake of diets typically rich in highly processed foods (with high fat content, low in complex carbohydrates and rich in refined sugars and salt), access to healthcare and pharmaceutical products, hygiene and sanitation conditions, reduced exposure to livestock, and increased population density. The classification was based on the information available on how populations included in the study differ on the above criteria and how the samples were reported in the original publications. While we acknowledge that this binary classification has evident limitations^62^, it enables insight into the association of person-to-person microbiome transmission with host lifestyle.

#### Newly sequenced metagenomic datasets

##### Argentina dataset

A total of 14 mothers (16–37 years old) and 13 of their infants below 1 year of age in rural areas in Argentina (villages of Villa Minetti, Esteban Rams, Pozo Borrado, Las Arenas, Cuatro Bocas, Logroño, Montefiore and Belgrano; Santa Fe province; Supplementary Table 2)—considered here as a non-westernized population—were enroled in the study. DNA was extracted from faecal samples using the QIAamp DNA stool kit (Qiagen) following the manufacturer’s instructions. Sequencing libraries were prepared using the Nextera DNA Flex Library Preparation Kit (Illumina), following the manufacturer’s guidelines. Sequencing was performed on the Illumina NovaSeq 6000 platform following manufacturer’s protocols.

##### Colombia dataset

A total of 12 mothers (15–40 years old) and 12 of their infants below 6 months of age from communities of the Wayúu ethnic group from the Caribbean Region in Colombia (communities of Etkishimana, Koustshachon, Paraiso, Invasión, Tocomana, Warruptamana and Wayawikat; Supplementary Table 2)—considered here as a non-westernized population—were enroled in the study. DNA from stool samples was extracted using the Master-Pure DNA extraction Kit (Epicentre) following the manufacturer’s instructions with the following modifications: samples were treated with lysozyme (20 mg ml^−1^) and mutanolysin (5 U ml^−1^) for 60 min at 37 °C and a preliminary step of cell disruption with 3-μm diameter glass beads during 1 min at 6 m s^−1^ by a bead beater FastPrep 24-5G Homogenizer (MP Biomedicals). Purification of the DNA was performed using DNA Purification Kit (Macherey–Nagel) according to manufacturer’s instructions. DNA concentration was measured using Qubit 2.0 Fluorometer (Life Technologies) for further analysis. Sequencing libraries were prepared using the Nextera DNA Flex Library Preparation Kit (Illumina), following the manufacturer’s guidelines. Sequencing was performed on the Illumina NovaSeq 6000 platform following manufacturer’s protocols.

##### China_1 dataset

A total of 116 nonagenarians and centenarians (97 female, 19 male, 94–105 years old) and 231 of their offspring (79 female, 152 male, 50–85 years old) in the city of Qidong (Jiangsu province, China) were enroled (considered here as a westernized population)^63^. All participants were free of major illnesses at the time of inclusion. Fresh stool samples were collected at the Shanghai Tenth Hospital, and stored at −20 °C upon collection. DNA was extracted using the EZNA Stool DNA Kit (Omega Bio-tek) following manufacturer’s instructions. DNA integrity and size were evaluated by 1% agarose gel electrophoresis, and DNA concentrations determined with NanoDrop (Thermo Fisher Scientific). DNA libraries were constructed according to the TruSeq DNA Sample Prep v2 Guide (Illumina), with 2 μg of genomic DNA and an average insert size of 500 bp. Library quality was evaluated with a DNA LabChip 1000 Kit (Agilent Technologies). Sequencing was conducted on an Illumina HiSeq 4000 platform with a 150 bp paired-end read length.

##### China_2 dataset

A total of 8 mothers and 19 infants below 1 year of age in a rural population in China (Bin county, Shaanxi province, northwest China) were enroled as part of a larger study (ClinicalTrials.gov NCT02537392); they were considered here as a non-westernized population. DNA was extracted with the QIAamp Fast DNA Stool Mini Kit (Qiagen), and precipitated with ethanol. Sequencing libraries were prepared using the Nextera DNA Flex Library Preparation Kit (Illumina), following the manufacturer’s guidelines. Sequencing was performed on the Illumina NovaSeq 6000 platform following manufacturer’s protocols.

##### Guinea-Bissau dataset

Samples from 342 volunteers (0–85 years old) in 74 households in the island of Bubaque (Bijagos Archipelago, Guinea-Bissau)—considered here as a non-westernized population—were collected and DNA extracted as part of a previous study^64^. In brief, samples were frozen at −20 °C at a reference laboratory. After homogenization and washing, DNA was extracted using the DNeasy PowerSoil PRO kit (Qiagen) with custom modifications^64^. Sequencing libraries were prepared using the Nextera DNA Flex Library Preparation Kit (Illumina), following the manufacturer’s guidelines. Sequencing was performed on the Illumina NovaSeq 6000 platform following manufacturer’s protocols.

##### Italy_1 dataset

A total of 4 mothers (37–46 years old) and their 8 children (0–2 years old) were enroled at the Santa Chiara Hospital in Trento, Italy; they were considered here as a westernized population. Mother stool samples were collected during or shortly after the delivery by the hospital staff, using faecal material collection tubes (Sarstedt). Infant stool samples were collected by the mothers, frozen at −20 °C upon collection and moved to a −80 °C facility within a week. 48 samples were collected in total (Supplementary Table 2). DNA was extracted using the PowerSoil DNA Isolation Kit (MoBio Laboratories), as described in the HMP protocol (Human Microbiome Project Consortium)^65^, with addition of a preliminary heating step (65 °C for 10 min, 95 °C for 10 min). DNA was recovered in 10 mM Tris pH 7.4 and quantified using the Qubit 2.0 (Thermo Fisher Scientific) fluorometer per the manufacturer’s instructions. Sequencing libraries were prepared using the NexteraXT DNA Library Preparation Kit (Illumina), following the manufacturer’s guidelines. Sequencing was performed on the Illumina HiSeq 2500 platform.

##### Italy_2 dataset

A total of 19 mothers (30–47 years old) and 37 healthy children (0–11 years old) were enroled at the IRCCS Istituto Giannina Gaslini in Genoa, Italy as part of a larger study, considered here as a westernized population. Stool samples were collected in DNA/RNA shield faecal collection tubes (Zymoresearch) and stored at −80 °C until DNA extraction. DNA extraction was performed with the DNeasy PowerSoil Pro Kit (Qiagen) according to the manufacturer’s procedures. DNA concentration was measured using the NanoDrop spectrophotometer (Thermo Fisher scientific) and stored at −20 °C. Sequencing libraries were prepared using the NexteraXT DNA Library Preparation Kit (Illumina), following the manufacturer’s guidelines. Sequencing was performed on the Illumina NovaSeq 6000 platform following manufacturer’s protocols.

##### USA dataset

A total of 1,929 saliva samples from 646 families in the NY Genome Center Cohort of the SPARK collection (Western IRB (https://www.wcgirb.com/), protocol tracking number: WIRB20151664, considered here as a westernized population) were included in the analysis, consisting of 640 mother samples (22–55 years old), 631 father samples (23–67 years old), and 658 samples from normally developing offspring (0–18 years old). Saliva was collected using the OGD-500 kit (DNA Genotek), and DNA was extracted using a Chemomagic MSM1/360 DNA extraction instrument and eluted into 110ul of TE buffer at PreventionGenetics (Marshfield). Sequencing libraries were prepared with the Illumina DNA PCR-Free Library Prep kit (Illumina), following the manufacturer’s guidelines. Sequencing was performed on the Illumina NovaSeq 6000 platform using S2/S4 flow cells and following manufacturer protocols.

### Metagenome pre-processing and quality control

Newly sequenced stool samples were pre-processed using the pipeline described at https://github.com/SegataLab/preprocessing. Shortly, metagenomic reads were quality-controlled and reads of low quality (quality score <Q20), fragmented short reads (<75 bp), and reads with >2 ambiguous nucleotides were removed with Trim Galore (v0.6.6). Contaminant and host DNA was identified with Bowtie2 (v2.3.4.3)^66^ using the -sensitive-local parameter, allowing confident removal of the phiX 174 Illumina spike-in and human-associated reads (hg19 human genome release). Remaining high-quality reads were sorted and split to create standard forward, reverse and unpaired reads output files for each metagenome.

Newly sequenced saliva samples were pre-processed using a custom version of the pipeline described in https://github.com/SegataLab/preprocessing. Shortly, metagenomic reads were quality-controlled, removing reads of low quality (quality score <Q20), fragmented short reads (<75 bp), and reads with >2 ambiguous nucleotides. Contaminant and host DNA was identified with Bowtie2 (v2.3.5.1)^66^ in ‘end-to-end’ global mode, allowing confident removal of human-associated reads (hg19). Remaining high-quality reads were sorted and split to create standard forward, reverse and unpaired reads output files for each metagenome.

Read statistics of stool and saliva samples (number of reads, number of bases, minimum and median read length per sample) are detailed in Supplementary Table 2. Metagenomes with ≥3 million reads were included in the analysis (*n* = 7,646 stool, *n* = 2,069 oral), while metagenomes with insufficient sequencing depth were excluded (*n* = 97 stool, *n* = 0 oral).

### Expanded SGB database

A custom database containing 160,267 MAGs and 75,446 isolate sequencing genomes was retrieved from ref. ^30^, and expanded with 184 MAGs from the Italian mother–infant dataset^9^ expanded in the current study, 1,439 MAGs from Italian centenarians^67^, 3,584 MAGs obtained from stool samples of individuals in non-westernized populations^34^, 2,985 MAGs from stool samples of non-human primates^68^, 20,404 MAGs from cow rumen^69^, 14,097 MAGs from mouse samples^70–83^, 1,235 MAGs from termites (PRJNA365052, PRJNA365053, PRJNA365054, PRJNA365049, PRJNA365050, PRJNA365051, PRJNA405700, PRJNA405701, PRJNA405702, PRJNA405782, PRJNA405783, PRJNA366373, PRJNA366374, PRJNA366375, PRJNA366251, PRJNA405703, PRJNA366252, PRJNA366766, PRJNA366357, PRJNA366358, PRJNA366361, PRJNA366362, PRJNA366363, PRJNA366255, PRJNA366256, PRJNA366257, PRJNA366253, PRJNA405704, PRJNA366254 and PRJNA405781), 7,760 MAGs available from a previous catalogue^84^, 2,137 MAGs from NCBI GenBank, and 63,142 reference genomes from NCBI GenBank (see https://github.com/SegataLab/MetaRefSGB for details). MAGs from the Italian mother–infant dataset, and those of non-human hosts were assembled using MEGAHIT^85^, while those of the Italian centenarian dataset and non-westernized populations were assembled with metaSPAdes^86^, using default parameters in both cases.

For the newly added MAGs we employed the following protocol on the metagenomic assemblies. Assembled contigs longer than 1,500 nucleotides were binned into MAGs using MetaBAT2^87^. Quality control of all genomes was performed with CheckM version 1.1.3 (ref. ^88^), and only medium- and high-quality genomes (completeness ≥50% and contamination ≤5%) were included in the database. Prokka version 1.12 and 1.13 (ref. ^89^) were used to annotate open reading frames of the genomes. Coding sequences were then assigned to a UniRef90 cluster^90^ by performing a Diamond search (version 0.9.24)^91^ of the coding sequences against the UniRef90 database (version 201906) and assigning a UniRef90 ID if the mean sequence identity to the centroid sequence was above 90% and covered more than 80% of the centroid sequence. Protein sequences that could not be assigned to any UniRef90 cluster were de novo clustered using MMseqs2^92^ within SGBs following the Uniclust90 criteria^93^.

Genomes were clustered into species-level genome bins (SGBs) spanning ≤5% genetic diversity, and those to genus-level genome bins (GGBs, 15% distance) and family-level genome bins (FGBs, 30% distance), as described in ref. ^30^. MAGs were assigned to SGBs by applying ‘phylophlan_metagenomic’, a subroutine of PhyloPhlAn 3 (ref. ^94^), which uses Mash^95^ to compute the whole-genome average nucleotide identity among genomes. When no SGB was below 5% genetic distance to a genome, new SGBs were defined, based on the average linkage assignment and hierarchical clustering (allowing a 5% genetic distance among genomes in the dendrogram). The same procedure was followed to assign SGBs to novel GGBs and FGBs when those were not yet defined.

### Taxonomic assignment of SGBs and definition of kSGBs and uSGBs

SGBs containing at least one reference genome (kSGBs) were assigned the taxonomy of the reference genomes following a majority rule, up to the species level. SGBs with no reference genomes (uSGBs) were assigned the taxonomy of its corresponding GGB (up to the genus level) if this contained reference genomes, and of its corresponding FGB (up to the family level) if the latter contained reference genomes. If no reference genomes were present in the FGB, a phylum was assigned based on the majority rule applied on up to 100 closest reference genomes to the MAGs in the SGB as provided by ‘phylophlan_metagenomic’. Taxonomic assignment of SGBs profiled at strain level in this study can be found in Supplementary Tables 3 and 4.

### Species-level profiling of metagenomic samples

Species-level profiling was performed on all the 9,715 samples with MetaPhlAn 4 (refs. ^38,39^) with default parameters and the custom SGB database. uSGBs with less than 5 MAGs were discarded as potential assembly artefacts or chimeric sequences and unlikely to reach the prevalence thresholds in the profiling. SGB core genes were defined as open reading frames in an existing UniRef90 or in a de novo clustered gene family (following the Uniclust90 clustering procedure^93^) present in at least half of the genomes (that is, ‘coreness’ 50%) of the SGB. Core genes were further optimized by selecting the highest coreness threshold that allowed retrieval of at least 800 core genes. Core genes of each SGBs were then screened to identify marker genes by checking their presence in other SGBs. This was done by a procedure that first divided core genes into fragments of 150 nt and then aligned the fragments against the genomes of all SGBs using Bowtie2 (version 2.3.5.1; -sensitive option)^66^. Marker genes were defined as core genes with no fragments found in at least 99% of the genomes of any other SGB. For SGBs with less than 10 marker genes, conflicts were defined as occurrences of more than 200 core genes of an SGB in more than 1% of genomes of another SGB, and conflict graphs were generated by retrieving all conflicts for that SGB. Each conflict graph was processed iteratively, retrieving all the possible merging scenarios, in order to get the optimal merges for the conflict that both minimize the number of merged SGBs and maximize the number of markers retrieved. Finally, for each SGB, a maximum of 200 marker genes were selected based first on their uniqueness and then on their size (bigger first), and SGBs still with less than 10 markers were discarded. Merged gut and oral SGBs (SGB_group) can be found in Supplementary Tables 3 and 4, respectively. The resulting 3.3M marker genes (189 ± 34marker genes per SGB(mean ± s.d.)) were used as a new reference database for MetaPhlAn and StrainPhlAn profiling.

### Strain-level profiling of metagenomic samples

Strain profiling was performed with StrainPhlAn4^38,39^ using the custom SGB marker database, with parameters “marker_in_n_samples 1 -sample_with_n_markers 10 –phylophlan_mode accurate -mutation_rates”. To reduce noise, only SGBs detected in ≥20 samples and at least 10% of samples in a dataset with ≥10 markers (-print_clades_only argument in StrainPhlAn) were selected for strain-level profiling (*n* = 646 and *n* = 252 SGBs in stool and oral samples respectively). The total of 200 marker genes was available for the majority of SGBs (*n* = 481/646 gut SGBs and *n* = 148/252 oral SGBs). The average coverage across SGBs was 1.3×. For the SGBs potentially derived from fermented foods, sequences of MAGs assembled in ref. ^40^ were added using parameter “-r”. Compared to an assembly based approach (high-quality MAGs defined as >90% completeness and <5% contamination; assembly method reported in the section “Expanded SGB database” above), strain-level profiling with StrainPhlAn allowed strain-sharing assessment among species in many more samples (median of 355 strain-level profiles per SGB and interquartile range (IQR) = [185, 806] versus median of 69 high-quality MAGs per SGB and IQR = [7, 60]).

### Detection of strain-sharing events

To detect strain-sharing events, we first set SGB-specific normalized phylogenetic distance (nGD) thresholds that optimally separated same-individual longitudinal strain retention (same strain) from unrelated-individual (different strain) nGD distributions in five published stool metagenomic datasets from four different countries (Germany, Kazakhstan, Spain and United States) on three continents^20,22,27,28,31^. nGDs were calculated as leaf-to-leaf branch lengths normalized by total tree branch length in phylogenetic trees produced by StrainPhlAn, which are built on marker gene alignments on positions with at least 1% variability. For SGBs detected in at least 50 pairs of same-individual stool samples obtained no more than 6 months apart (*n* = 145 SGBs; the two samples for a certain individual in which the species could be profiled at the strain level and that were closest in time were selected), nGD thresholds were defined based on maximizing Youden’s index, and limiting at 5% the fraction of unrelated individuals to share the same strain as a bound on a false discovery rate (Extended Data Fig. 3). The assumption of frequent strain persistence in an individual for at least 6 months is supported by the distribution of phylogenetic distances in the longitudinal sets: for all species this has a peak at nGD approaching 0 (Extended Data Fig. 3), notably higher than that observed for inter-individual sample comparisons. For SGBs detected in less than 50 same-individual close pairs (*n* = 501) and in oral samples (*n* = 252), for which species-specific nGD cannot be reliably estimated, the nGD corresponding to the 3rd percentile of the unrelated individual nGD distribution was used. This value is the median percentile of the inter-individual nGD distribution corresponding to the nGD maximizing the Youden’s index of SGBs with at least 50 same-individual comparisons. The three sets of thresholds are thus three technical definitions of the same principle—that is, the individual specificity and the persistence of strains in the gut microbiome, and did not lead to significant differences in nGD values (Kruskal–Wallis test, *χ*^2^ = 2.34, *P* = 0.31; Extended Data Fig. 10a). nGD thresholds also did not significantly differ by phylum (Extended Data Fig. 10b), and those set in stool and oral samples were similar (median nGD difference = 0.006). If not limiting at 5% the fraction of unrelated individuals to share the same strain as a bound on a false discovery rate, the resulting percentile would only be of a median of 8.2% (range = [5.2–22.3%]) on these 38 SGBs (Supplementary Table 4). When using single metagenomic datasets instead of the five datasets we included to set the strain identity thresholds, often not enough longitudinal samples were available (<50 same-individual pairs) and some variation was observed (Extended Data Fig. 10c), which supports the use of the largest set of samples available.

Overall, the median SNV rate nGD thresholds corresponded to is 0.005, below the estimated >0.1% sequencing error rate by Illumina HiSeq and NovaSeq platforms^96^ (Supplementary Table 4). The nGD thresholds correspond to a SNV rate of 0 for some SGBs (*n* = 16 out of 646—that is, 2.5%), mostly those encompassing very low genetic variation (for example, *B. animalis* SGB17278). In SGB trees containing MAGs of microorganisms obtained from fermented foods, we identified and discarded any strains with high similarity (≤0.0015 SNV rate as determined by PhyloPhlAn 3 (https://github.com/biobakery/phylophlan/wiki#mutation-rates-table)—that is, the number of positions that have nucleotide differences divided by the length of the alignment) to food MAGs (Supplementary Table 6). For *B. animalis* (SGB17278), 62 strains profiled in 7 public mouse metagenome datasets^73,75,97–101^ were added to better assess its phylogenetic diversity. The trees produced by StrainPhlAn together with the SGB-specific nGD thresholds were used in StrainPhlAn4’s strain_transmission.py script (-threshold argument) (https://github.com/biobakery/MetaPhlAn/blob/master/metaphlan/utils/strain_transmission.py). Pairs of strains with pairwise nGD below the strain identity threshold were defined as strain-sharing events. Centred nGD is defined as the nGD divided by the median nGD in the phylogenetic tree. We opted for strain identity thresholds based on phylogenetic distances in contrast to SNV rates due to (1) the rather low coverage that we obtain for species in metagenomic samples even after passing our sequencing depth threshold (mean coverage = 7.2×, median = 0.69 and IQR = [0.14, 3.09]) that would add noise especially to SNV rate estimations; (2) the limited length of the marker gene alignment of some SGBs (mean trimmed alignment length = 74,348 nt, median = 70,879 and IQR = [42,513, 104,347]) that would make SNV rates rather unreliable; and (3) the valuable information on evolutionary models (for example, distinguishing synonymous from non-synonymous nucleotide changes) that is provided by phylogenetic trees.

We compared the new species-specific strain identity thresholds with the nGD = 0.1 threshold (that is, considering the lowest 10% phylogenetic distances to be between the same strains) used in some previous publications and StrainPhlAn versions prior to version 4 (refs. ^9,32,102^). We found that while the previous threshold would produce a median 44% mother–infant strain-sharing rate—in contrast to the 50% strain-sharing rate we obtain here—the novel method yields a lower strain-sharing rate between infants and unrelated mothers, which are likely to be false positives: 3.5% versus 4%. This supports the better performance of the species-specific strain identity thresholds as they detect—at the same time—more strain-sharing events between matched mothers and infants and fewer strain-sharing events between unrelated mother–infant pairs.

To assess the reproducibility of the species-specific strain identity thresholds on additional unrelated data, we used independent datasets of patients undergoing faecal microbiome transplantation (FMT). As we used the publicly available metagenomic cohorts with no intervention and longitudinal sampling^20,22,27,28,31^ to set the species-specific thresholds, we used for validation the completely independent FMT datasets as a distinct setting in which strain transmission can be expected. In FMT, part of the strains from a healthy donor are successfully transferred to a patient, while some strains from the donor’s original sample remain after the intervention. We included 1,371 samples from 25 different cohorts of patients undergoing FMT^103–123^ that were analysed as part of a meta-analysis^124^. In this evaluation, similar to what we did in the set of longitudinal samples, we assessed the separation between the distribution of the nGD distances of strains from the same SGB in the two following situations: (1) the strains are from samples of the same individual or from a FMT donor and their recipient after the FMT, and (2) the strains are from samples belonging to different FMT triads (defined by the samples from the donor, those of the patient before FMT, and those of the patients after FMT). We performed this analysis for each of the 95 SGBs of our set that were also profiled in the Ianiro et al study. We considered as true positives pairwise phylogenetic distance (nGD) values between samples in (1) that were below the species-specific strain identity threshold (defined on the independent longitudinal datasets), false positives as those from (2) that were below the threshold, true negatives as those from (2) above the threshold, and false negatives as those from (1) above the threshold. We found that StrainPhlAn4 with the species-specific strain identity thresholds defined here performed very well in distinguishing strains in the same individual or FMT triad from different strains in different FMT triads: median recall = 0.97 and IQR = [0.95,0.99], precision = 0.72 [0.67,0.82], *F*-score = 0.97 [0.96,0.98] (Supplementary Table 35).

### Assessment of person–person strain-sharing rates and SGB transmissibility

**Extended Data Fig. 6:**
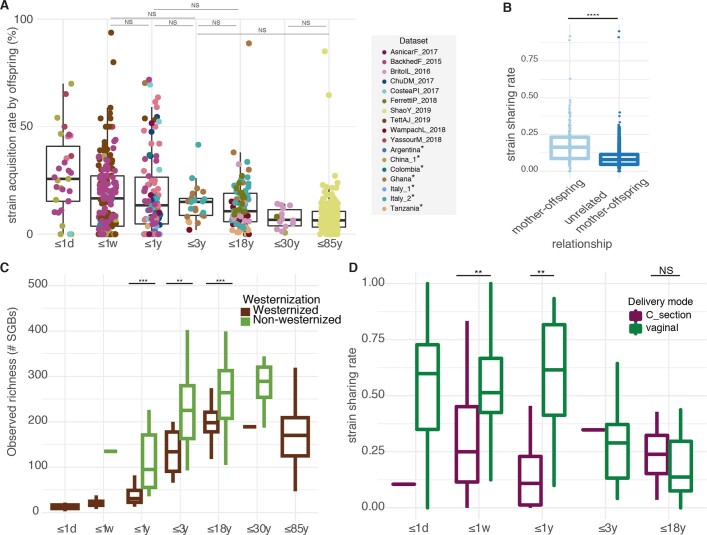
Mother to offspring gut microbiome transmission. **A**) Strain acquisition rates by the offspring tend to decrease as a function of the offspring’s age. Strain acquisition rates by the offspring are defined as the proportion of strains profiled in the offspring that are shared with their mother, computed in 17 datasets from 14 different countries across pre-defined age categories. Kruskal-Wallis test, Chi2=65, P = 3.57e-12, Post-hoc Dunn tests, NS corresponds to Padj≥0.05, all other comparisons are significant (Table S10). Boxes: lower and upper quartiles, middle line: median; whiskers: 1.5 × IQR. Novel datasets are highlighted with asterisks. **B**) Strain sharing rates between senior individuals and their non-cohabiting mothers as compared to strain sharing rates between unrelated mother-offspring pairs. Wilcoxon rank-sum test, N = 17,177, r = 0.09, P = 4.1e-35. Boxes: lower and upper quartiles, middle line: median; whiskers: 1.5 × IQR. **C**) Observed richness (number of SGBs detected with MetaPhlAn) in age categories of offspring from Westernized as compared to non-Westernized populations. Wilcoxon rank-sum tests, N = 721, ***Padj <0.001 and **Padj<0.01, Table S11. Boxes: lower and upper quartiles, middle line: median; whiskers: 1.5 × IQR. **D**) Mother-offspring strain sharing rates in age categories of offspring delivered by C-section as compared to vaginally-delivered offspring. Wilcoxon rank-sum tests, **Padj<0.01, NS Padj≥0.05, Table S14. Boxes: lower and upper quartiles, middle line: median; whiskers: 1.5 × IQR.

**Extended Data Fig. 7:**
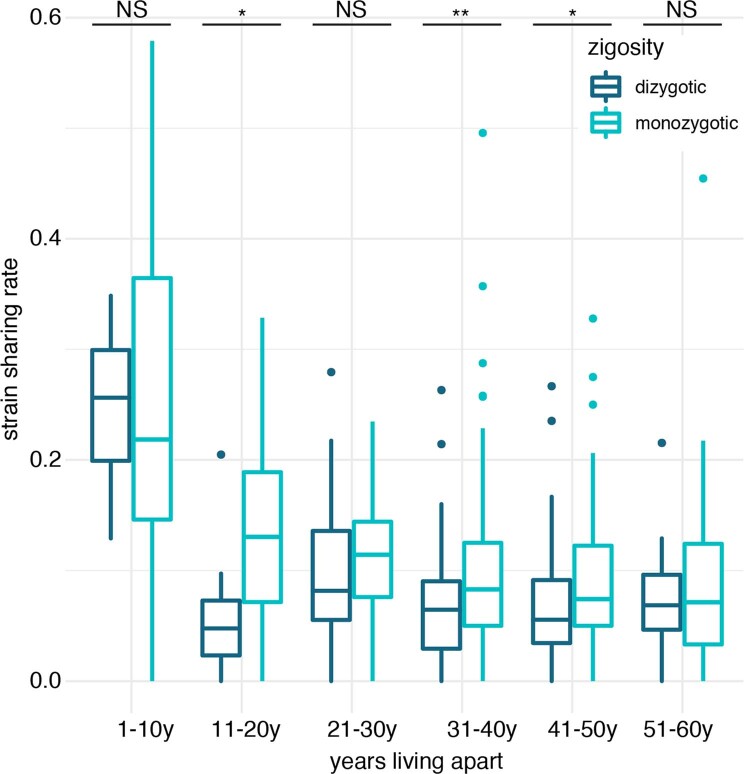
Gut microbiome strain sharing among adult twins. Dizygotic and monozygotic twin gut microbiome strain sharing rates after decades since cohabitation. Wilcoxon rank-sum tests, N = 708, **Padj<0.01, *Padj<0.05, NS Padj≥0.05, Table S19. Boxes: lower and upper quartiles, middle line: median; whiskers: 1.5 × IQR.

**Extended Data Fig. 8:**
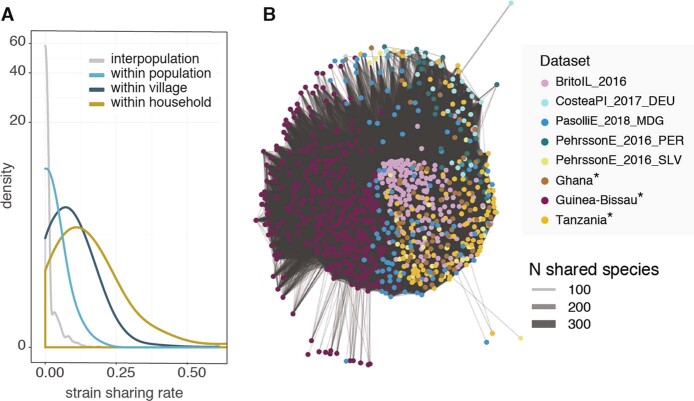
Gut microbiome species and strain sharing among individuals. **A**) Density distributions of gut microbiome strain sharing rates between household members (within household), individuals in different households in the same village (within village), individuals in different villages of the same population (within population), and in different populations (interpopulation). **B**) Gut microbiome species sharing unsupervised network of household datasets. Line width is proportional to the number of shared species. Only connections with ≥50 shared species are shown.

**Extended Data Fig. 9:**
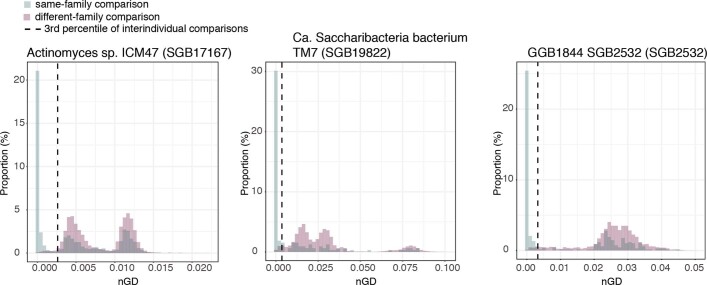
Highly-transmitted SGBs in oral samples. Same-family (green) to different-family (purple) genetic distance comparisons for the three SGBs consistently and significantly highly-transmitted in oral metagenomes. Strain identity thresholds were set as the 3rd percentile of the unrelated individual comparisons (dashed line).

**Extended Data Fig. 10:**
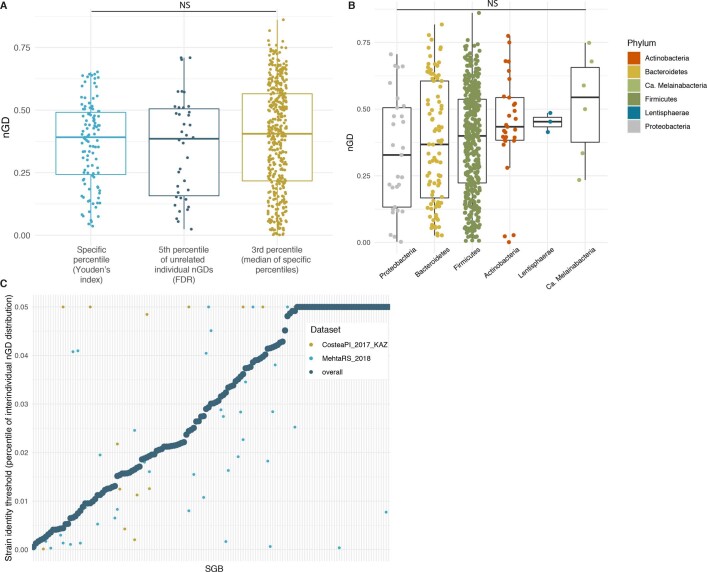
Assessment of strain identity thresholds. **A**) Centred nGD (normalised phylogenetic distance divided by the median nGD of the phylogenetic tree) used as a threshold for strain identity (corresponding to the percentiles of interindividual distributions) by strain definition used, for the 646 SGBs profiled in stool samples. The different percentiles do not result in significant differences in nGD values (Kruskal-Wallis test, Chi2=2.34, P = 0.31). Boxes: lower and upper quartiles, middle line: median; whiskers: 1.5 × IQR. **B**) Distribution of centred nGD thresholds (normalised phylogenetic distance divided by the median nGD of the phylogenetic tree) by phylum, showing lack of statistically-significant association (Kruskal-Wallis test, Chi2=6.6, P = 0.25). Boxes: lower and upper quartiles, middle line: median; whiskers: 1.5 × IQR. **C**) Strain identity thresholds (percentile of interindividual nGD distribution) calculated for each of the SGBs prevalent in longitudinal datasets (N = 145 SGBs profiled in at least 50 same-individual pairs) calculated on single datasets compared to the threshold used in the study (determined on all samples).

Person-to-person strain-sharing rates were calculated as the number of strains shared between two individuals divided by the number of shared SGBs profiled by StrainPhlAn (number of shared strains/number of shared SGBs). When multiple samples were available for an individual, detection of strain or SGB sharing at any time point was considered as the strain or SGB was shared. For a robust calculation, person-to-person strain-sharing rates were only assessed when at least ten SGBs were shared between two individuals. The same calculation was used to assess same-individual strain retention between two time points in longitudinal datasets. Strain acquisition rates by the offspring (Extended Data Fig. 6a) were defined as the proportion of strains profiled in the offspring that were shared with the mother, thus putatively originating from her. For a robust calculation, strain acquisition rates by the offspring were only assessed when at least ten SGBs were shared between the mother and the offspring. As StrainPhlAn^36,38,39^ profiles the dominant strain for each species, the total number of strains shared between two samples ranges between 0 and the total number of shared profiled SGBs, whereas strain-sharing rates and strain acquisition rates by the offspring are bound between 0 and 1.

SGB transmissibility was defined as the number of strain-sharing events detected for an SGB divided by the total potential number of strain-sharing events based on the presence of a strain-level profile by StrainPhlAn4. When multiple samples were available for an individual, detection of strain sharing at any time point was considered as the strain was shared. For a robust calculation, SGB transmissibility was only assessed on SGBs with at least ten potential strain-sharing events in multiple datasets, and with at least three potential strain-sharing events for single dataset calculations. To assess concordance of SGB transmissibility among datasets, Spearman’s correlations (cor.test function in R (https://www.R-project.org/)) were performed between datasets with at least ten SGBs with assessed transmissibility. Highly transmitted SGBs were defined as those with SGB transmissibility >0.5 and significantly higher within-group than among-group transmissibility (Chi-squared tests, *P*_adj_ < 0.05). We found no significant association between SGB transmissibility and the length of the trimmed alignment (Spearman’s test, *ρ* = 0.06, *P* = 0.13).

We assessed strain sharing across three main transmission modes: mother–infant (defined between mother and their offspring up to one year of age), household (defined as between cohabiting individuals), and intra-population (defined as that between non-cohabiting individuals in a population with no evidence of kinship).

### Species-level beta diversity and ordination

For the appropriate analysis of microbiome compositional data, species-level abundance matrices obtained by MetaPhlAn were centred log ratio-transformed using the codaSeq.clr function in the CoDaSeq R package (v0.99.6)^125^, using the minimum proportional abundance detected for each taxon for the imputation of zeros. A principal component analysis plot on Aitchison distance was produced with the ordinate and plot_ordination function in phyloseq (v1.28.0)^126^, using one randomly selected sample per individual (*n* = 4,840 gut samples, *n* = 2,069 oral samples). To compare species-level similarity to strain-sharing rates, beta diversity metrics (Aitchison distance, Bray–Curtis dissimilarity, and Jaccard binary distance) computed with the vegan R package (v2.5–7) were converted to similarity indices (1 − (distance or dissimilarity)).

### Strain–sharing networks

Unsupervised networks based on shared strains and species were visualized with R packages ggraph (v2.0.5), igraph (v1.2.6)^127^, and tidygraph (v1.2.0) with stress layout, showing connections with ≥5 shared strains or ≥50 shared species (edges) among individuals (nodes).

### Annotation of species phenotypic traits

Experimentally determined bacterial phenotypes were fetched from the Microbe Directory v2.0 (ref. ^128^), and matched to kSGBs by NCBI taxonomic identifiers. Phenotypic traits that have previously been hypothesized to be linked with species transmissibility^3^ were predicted for all SGBs using Traitar (version 1.1.12)^60^ on the 50% core genes (genes present in 50% of genomes available in the expanded SGB database). Only annotations for which the phypat and the phypat + PGL classifiers (the second including additionally evolutionary information on phenotype gains and losses) annotations matched were kept. Associations between SGB transmissibility and microorganism phenotypes were assessed with Wilcoxon rank-sum tests on the 25% most transmissible SGBs as compared to the 25% least transmissible ones.

### Statistical analysis

Statistical analyses and graphical representations were performed in R using packages vegan (version 2.5–7), phyloseq (v1.28.0)^126^, QuantPsyc (v1.5), ggplot2 (v3.3.3), ggpubr (v0.4.0) and corrplot (v0.84). Correction for multiple testing (Benjamini–Hochberg procedure, *P*_adj_) was applied when appropriate and significance was defined at *P*_adj_ < 0.05. All tests were two-sided except where specified otherwise. The association between metadata variables and distance matrices was assessed by PERMANOVA with the adonis function in vegan. Differences between two groups were assessed with Wilcoxon rank-sum tests. For more than two groups, the Kruskal–Wallis test with post hoc Dunn tests was used. Correlations were assessed with Spearman’s tests. To assess correlations between variables while partialling out potential confounders, GLMs were fitted with the glm R function (Gaussian, link = identity). Standardized GLM regression coefficients were calculated using the lm.beta R function (QuantPsyc R package). The significance was assessed by performing log likelihood (Chi-squared) tests on nested GLMs.

### Ethical compliance

All study procedures are compliant with all relevant ethical regulations. The procedures were performed in compliance with the Declaration of Helsinki. Ethical approval of the Argentina cohort was granted by the Ethics and Safety committee (CEySTE), CCT Santa Fe, Argentina (29112019). The Colombia cohort was approved by the Research Bioethics committee, Universidad Metropolitana, Colombia (NIT 890105361-5). The China_1 dataset research protocol was approved by the Ethics Committee of Shanghai Tenth Hospital, Tongji University School of Medicine (SHSY-IEC-pap-18-1), and China_2 was approved by the Ethics committee of the Health Science Center, Xi’an Jiaotong University, China (2016-114). The Guinea-Bissau study was approved by the Health Ethics National Committee (Comitê Nacional da Ética na Saude), Ministry of Public Health, Guinea-Bissau (076/CNES/INASA/2017) and by the London School of Hygiene and Tropical Medicine Ethics Committee (reference number 22898). The Italy_1 dataset research protocol was approved by the Ethics Committee of Santa Chiara Hospital, Trento, Italy (51082283, 30 July 2014) and the Ethics Committee of the University of Trento, Italy, and Italy_2 by the Liguria Regional Ethics Committee, Italy (006/2019). Ethical approval for the USA dataset was granted by Western IRB (https://www.wcgirb.com/), with protocol tracking number WIRB20151664. Written informed consent was obtained from all adult participants and from parents of non-adult participants.

### Reporting summary

Further information on research design is available in the Nature Portfolio Reporting Summary linked to this article.

## Online content

Any methods, additional references, Nature Portfolio reporting summaries, source data, extended data, supplementary information, acknowledgements, peer review information; details of author contributions and competing interests; and statements of data and code availability are available at 10.1038/s41586-022-05620-1.

## Supplementary information

Supplementary Information This file contains a guide to Supplementary Tables 1–35 (tables supplied separately) and a link to a tutorial describing the procedure followed to assess strain sharing.

Reporting Summary

Supplementary Tables Supplementary Tables 1–35: see Supplementary Information document for full descriptions.

## Data Availability

Shotgun metagenomics sequencing data of the Argentina, Colombia, China_2, Guinea-Bissau, Italy_1 and USA datasets are available at the European Nucleotide Archive under accession number PRJEB45799. The sequencing data of the China_1 dataset is available on the NCBI Sequence Read Archive database with accession PRJNA613947. The sequencing data of the Italy_2 dataset is on the NCBI Sequence Read Archive database with accession PRJNA716780. Metadata are available in Supplementary Table 2 and in the latest release of curatedMetagenomicData^61^.

## Code availability

All the software and thresholds developed and used in this study are available in the MetaPhlAn4 package^39^ (which includes StrainPhlAn4 and the script for strain transmission inference with the species-specific strain identity thresholds), available at http://segatalab.cibio.unitn.it/tools/metaphlan with the open-source code at https://github.com/biobakery/MetaPhlAn. It is also available via Bioconda (https://anaconda.org/bioconda/metaphlan) and PIP (https://pypi.org/project/MetaPhlAn). A tutorial describing the procedure we followed to assess strain sharing is available at https://github.com/biobakery/MetaPhlAn/wiki/Strain-Sharing-Inference.
