# Supplementary material for: The person-to-person transmission landscape of the gut and oral microbiomes
Source: Nature. 2023 Jan 18;614(7946):125–35. doi: 10.1038/s41586-022-05620-1 (PMC9892008; doi:10.1038/s41586-022-05620-1)
Supplement: Supplementary file 1 — This file contains a guide to Supplementary Tables 1–35 (tables supplied separately) and a link to a tutorial describing the procedure followed to assess strain sharing. [file 41586_2022_5620_MOESM1_ESM.pdf]

nature portfolio

https://doi.org/10.1038/s41586-022-05620-1

Supplementary information

The person-to-person transmission landscape of the gut and oral microbiomes

In the format provided by the authors and unedited

SUPPLEMENTARY INFORMATION GUIDE

The person-to-person transmission landscape of the gut and oral microbiomes

Mireia Valles-Colomer[1,*], Aitor Blanco-Míguez[1], Paolo Manghi[1], Francesco Asnicar[1], Leonard Dubois[1], Davide Golzato[1], Federica Armanini[1], Fabio Cumbo[1], Kun D. Huang[1], Serena Manara[1], Giulia Masetti[1], Federica Pinto[1], Elisa Piperni[2], Michal Punčochář[1], Liviana Ricci[1], Moreno Zolfo[1], Olivia Farrant[3], Adriana Goncalves[3], Marta Selma-Royo[1,4], Ana G. Binetti[5], Jimmy E. Becerra[6], Bei Han[7], John Lusingu[8], John Amuasi[9], Loredana Amoroso[10], Alessia Visconti[11], Claire M. Steves[11], Mario Falchi[11], Michele Filosi[1], Adrian Tett[1,12], Anna Last[3], Qian Xu[13,14], Nan Qin[13,14], Huanlong Qin[13], Jürgen May[15], Daniel Eibach[15], Maria Valeria Corrias[16], Mirco Ponzoni[16], Edoardo Pasolli[17], Tim D. Spector[11], Enrico Domenici[1,18], Maria Carmen Collado[4], Nicola Segata[1,2,*]

1. Department CIBIO, University of Trento, Trento, Italy.
2. Department of Experimental Oncology, IEO European Institute of Oncology IRCCS, Milan, Italy.
3. Clinical Research Department, Faculty of Infectious and Tropical Diseases, London School of Hygiene & Tropical Medicine, London, United Kingdom.
4. Institute of Agrochemistry and Food Technology, National Research Council (IATA-CSIC), Paterna, Valencia, Spain.
5. Instituto de Lactología Industrial (CONICET-UNL), Facultad de Ingeniería Química, Universidad Nacional del Litoral, Santa Fe, Argentina.
6. Grupo de Investigación Alimentación y Comportamiento Humano, Universidad Metropolitana - Barranquilla, Colombia.
7. School of Public Health, Health Science Center, Xi'an Jiaotong University, Xi'an, China.
8. National Institute for Medical Research, Tanga Centre, Tanzania.
9. Kumasi Centre for Collaborative Research in Tropical Medicine, Kwame Nkrumah University of Science and Technology, Ghana.
10. Oncology Unit, IRCCS Istituto Giannina Gaslini, Genoa, Italy.
11. Department of Twin Research & Genetic Epidemiology, King's College London, London, United Kingdom.
12. Centre for Microbiology and Environmental Systems Science, University of Vienna, Vienna, Austria.
13. Shanghai Tenth People's Hospital, Tongji University School of Medicine, Shanghai, China.
14. Realbio Genomics Institute, Shanghai, China.
15. Bernhard Nocht Institute for Tropical Medicine, Hamburg, Germany.
16. Laboratory of Experimental Therapies in Oncology, IRCCS Istituto Giannina Gaslini, Genoa, Italy.
17. Department of Agricultural Sciences, University of Naples "Federico II", Portici, Italy.
18. Centre for Computational and Systems Biology (COSBI), Microsoft Research Foundation, Rovereto, Italy.

*Correspondence: mireia.vallescolomer@unitn.it (MV-C) and nicola.segata@unitn.it (NS).

SUPPLEMENTARY TABLE LEGENDS

Table S1. Summary of the 9,715 samples included in the study by dataset.

Table S2. Metadata of the 9,715 samples included in the study.

Table S3. PERMANOVA on same vs different individual genetic distance separation for each prevalent gut SGB.

Table S4. List of profiled SGBs in stool samples, taxonomic classification, and strain identity thresholds.

Table S5. List of profiled SGBs in saliva samples, taxonomic classification, and strain identity thresholds.

Table S6. Exclusion of samples phylogenetically close (≤0.0015 SNV rate) to MAGs of microorganisms obtained from fermented foods.

Table S7. Post-hoc Dunn two-sided tests on gut microbiome person-to-person strain sharing rates across human relationships. Multiple testing-corrected P values (Benjamini–Hochberg procedure) are reported in the Padj column.

Table S8. Kruskal-Wallis tests and Post-hoc Dunn tests on gut strain- or species-level similarities (Bray-Curtis, Aitchison, and Jaccard) within households, in non-cohabiting individuals of the same population, and in individuals from different populations.

Table S9. Gut SGB mother-infant, household, and intrapopulation transmissibility.

Table S10. Post-hoc Dunn two-sided tests on mother-offspring gut strain sharing rates across offspring age categories. Multiple testing-corrected P values (Benjamini–Hochberg procedure) are reported in the Padj column.

Table S11. Wilcoxon rank-sum two-sided tests on stool microbiome observed richness (number of SGBs detected with MetaPhlAn) in age categories of offspring from wWsternized as compared to non-Westernized populations.

Table S12. Wilcoxon rank-sum two-sided tests on mother-offspring gut strain sharing rates in age categories of offspring from Westernized as compared to non-Westernized populations.

Table S13. Wilcoxon rank-sum two-sided tests on the number of shared strains between stool samples of mothers and offspring in age categories of offspring from Westernized as compared to non-Westernized populations.

Table S14. Wilcoxon rank-sum two-sided tests on mother-offspring gut strain sharing rates in age categories of offspring delivered by C-section as compared to vaginally-delivered offspring.

Table S15. Spearman's tests on consistency of gut SGB mother-infant transmissibility across datasets (two-sided).

Table S16. Gut SGB mother-infant transmissibility (Chi2 tests, two-sided).

Table S17. Wilcoxon rank-sum two-sided tests on gut strain sharing rates by cohabiting individuals as compared to non-cohabiting individuals.

Table S18. Post-hoc Dunn two-sided tests on gut strain sharing rates among family relationships. Multiple testing-corrected P values (Benjamini–Hochberg procedure) are reported in the Padj column.

Table S19. Wilcoxon rank-sum two-sided tests on gut strain sharing rates between monozygotic twins (MZ) as compared to dizygotic twins (DZ) by 10-year age categories.

Table S20. Gut SGB household transmissibility and twin transmissibility (Chi2 tests, two-sided).

Table S21. Spearman's tests on consistency of gut SGB household transmissibility across datasets (two-sided).

Table S22. Post-hoc Dunn two-sided tests on gut strain sharing rates within household members, within villages, within populations, and interpopulations. Multiple testing-corrected P values (Benjamini–Hochberg procedure) are reported in the Padj column.

Table S23. Wilcoxon rank-sum two-sided tests on gut strain sharing rates among non-cohabiting individuals in the same village as compared to among villages.

Table S24. Gut SGB intrapopulation transmissibility (Chi2 tests, two-sided).

Table S25. Spearman's tests on consistency of gut SGB intrapopulation transmissibility across datasets (two-sided).

Table S26. Kruskal-Wallis tests and Post-hoc Dunn tests on oral strain- or species-level similarities (Bray-Curtis, Aitchison, and Jaccard) within households, in non-cohabiting individuals of the same population, and in individuals from different populations.

Table S27. Post-hoc Dunn two-sided tests on mother- and father-offspring oral strain sharing rates across age categories. Multiple testing-corrected P values (Benjamini–Hochberg procedure) are reported in the Padj column.

Table S28. Post-hoc Dunn two-sided tests on oral strain sharing rates among family relationships.

Table S29. Post-hoc Dunn two-sided tests on oral mother- as compared to father-offspring strain sharing rates by age categories. Multiple testing-corrected P values (Benjamini–Hochberg procedure) are reported in the Padj column.

Table S30. Oral SGB mother-infant, household, and intrapopulation transmissibility.

Table S31. Oral SGB mother-infant transmissibility (Chi2 tests, two-sided).

Table S32. Oral SGB household transmissibility (Chi2 tests, two-sided).

Table S33. Spearman's one-sided tests on SGB transmissibility and their median relative abundance and prevalence in metagenomes.

Table S34. SGB predicted phenotypical traits.

Table S35. Reproducibility assessment of the SGB-specific strain identity thresholds in independent FMT datasets.

SUPPLEMENTARY TUTORIAL

A tutorial describing the procedure we followed to assess strain sharing is available at https://github.com/biobakery/MetaPhlAn/wiki/Strain-Sharing-Inference
