# Supplementary material for: The person-to-person transmission landscape of the gut and oral microbiomes
Source: Nature. 2023 Jan 18;614(7946):125–35. doi: 10.1038/s41586-022-05620-1 (PMC9892008; doi:10.1038/s41586-022-05620-1)
Supplement: Supplementary file 2 — Reporting Summary [file 41586_2022_5620_MOESM2_ESM.pdf]

nature portfolio

Corresponding author(s): Nicola Segata

Last updated by author(s): 15/11/2022

Reporting Summary

Nature Portfolio wishes to improve the reproducibility of the work that we publish. This form provides structure for consistency and transparency in reporting. For further information on Nature Portfolio policies, see our Editorial Policies and the Editorial Policy Checklist.

Statistics

For all statistical analyses, confirm that the following items are present in the figure legend, table legend, main text, or Methods section.

n/a	Confirmed	
☐	☒	The exact sample size (*n*) for each experimental group/condition, given as a discrete number and unit of measurement
☐	☒	A statement on whether measurements were taken from distinct samples or whether the same sample was measured repeatedly
☐	☒	The statistical test(s) used AND whether they are one- or two-sided<br>*Only common tests should be described solely by name; describe more complex techniques in the Methods section.*
☐	☒	A description of all covariates tested
☐	☐	A description of any assumptions or corrections, such as tests of normality and adjustment for multiple comparisons
☐	☒	A full description of the statistical parameters including central tendency (e.g. means) or other basic estimates (e.g. regression coefficient) AND variation (e.g. standard deviation) or associated estimates of uncertainty (e.g. confidence intervals)
☐	☒	For null hypothesis testing, the test statistic (e.g. *F*, *t*, *r*) with confidence intervals, effect sizes, degrees of freedom and *P* value noted<br>*Give P values as exact values whenever suitable.*
☒	☐	For Bayesian analysis, information on the choice of priors and Markov chain Monte Carlo settings
☐	☒	For hierarchical and complex designs, identification of the appropriate level for tests and full reporting of outcomes
☐	☒	Estimates of effect sizes (e.g. Cohen's *d*, Pearson's *r*), indicating how they were calculated

Our web collection on statistics for biologists contains articles on many of the points above.

Software and code

Policy information about availability of computer code

Data collection	No software was used for microbiome data collection.
Data analysis	Newly-sequenced stool samples were pre-processed using the pipeline described in https://github.com/SegataLab/preprocessing, which uses Trim Galore (v0.6.6) and Bowtie2 (v2.3.4.3).<br>Newly-sequenced saliva samples were pre-processed using a custom version of the same pipeline as described in the Methods section, which uses Bowtie2 (v2.3.5.1). The custom SGB database was retrieved and expanded as described in the Methods section, using MEGAHIT, metaSPAdes, MetaBAT2, CheckM (v1.1.3), Prokka (v1.12 and v1.138), Diamond (v0.9.24), and MMseqs2.<br>Taxonomic assignment of MAGs and SGBs was performed using PhyloPhlAn 3, which uses Mash.<br>Species-level profiling was performed with MetaPhlAn 4 with default parameters and the custom SGB database. Strain-level profiling was performed with StrainPhlAn 4 using the custom SGB database and parameters "marker_in_n_samples 1 --sample_with_n_markers 10 –phylophlan_mode accurate --mutation_rates".<br>Strain transmission was inferred with the strain_transmission.py script (-d and -threshold arguments, option --restrictive) in StrainPhlAn 4. Pairs of strain with pairwise genetic distance below the strain identity threshold were defined as strain sharing events.<br>Experimentally-determined bacterial phenotypes were fetched from the Microbe Directory (v2.0). Phenotypic traits were predicted using Traitar (v1.1.12).<br>Statistical analyses and graphical representations were performed in R using packages vegan (v2.5-7), phyloseq (v1.28.0), QuantPsyc (v1.5), CoDaSeq (v0.99.6), ggplot2 (v3.3.3), ggpubr (v0.4.0), corrplot (v0.84), ggraph (v2.0.5), igraph (v1.2.6), and tidygraph (v1.2.0).

For manuscripts utilizing custom algorithms or software that are central to the research but not yet described in published literature, software must be made available to editors and reviewers. We strongly encourage code deposition in a community repository (e.g. GitHub). See the Nature Portfolio guidelines for submitting code & software for further information.

Data

Policy information about availability of data

All manuscripts must include a data availability statement. This statement should provide the following information, where applicable:

- Accession codes, unique identifiers, or web links for publicly available datasets
- A description of any restrictions on data availability
- For clinical datasets or third party data, please ensure that the statement adheres to our policy

Shotgun metagenomics sequencing data of the Argentina, Colombia, China_2, Guinea-Bissau, Italy_1, and USA datasets are available at the European Nucleotide Archive under accession number PRJEB45799. The sequencing data of the China_1 dataset is available on the NCBI Sequence Read Archive database with accession PRJNA613947. The sequencing data of the Italy_2 dataset is on the NCBI Sequence Read Archive database with accession PRJNA716780. Metadata are available in Table S2 and in the latest release of curatedMetagenomicData.

Field-specific reporting

Please select the one below that is the best fit for your research. If you are not sure, read the appropriate sections before making your selection.

☒ Life sciences ☐ Behavioural & social sciences ☐ Ecological, evolutionary & environmental sciences

For a reference copy of the document with all sections, see nature.com/documents/nr-reporting-summary-flat.pdf

Life sciences study design

All studies must disclose on these points even when the disclosure is negative.

Sample size	No sample size calculation was performed prior to cohort recruitment. This is the first global study on person-to-person microbiota transmission and therefore no previous information on sample size estimates was available. Therefore, we included all datasets (a total of 9,715 samples from 31 human metagenomic datasets) with available metadata to enable assessment of microbiome transmission between healthy mothers and offspring, households, twin pairs, villages, and populations (i.e., cohabitation information), as well as within-subject strain retention (longitudinal datasets with two samples obtained with less than 6 months difference). 25 datasets were publicly available, three of which were expanded with 14 (FerrettiP_2018) 8, 32 (Ghana dataset) 21, and 61 (Tanzania dataset) 21 samples. In addition, eight datasets (total: 2,800 samples) were newly collected and sequenced in the context of this study.
Data exclusions	No data were excluded from the analyses.
Replication	The strain identity thresholds identified were validated in independent datasets (1,371 samples from 25 different cohorts of patients undergoing FMT). Not applicable for the other sections, as all data available was included in the analyses.
Randomization	Not applicable: this was a cross-sectional study, not a randomized study. No intervention was performed on subjects, and therefore no random allocation into groups.
Blinding	Not applicable: this was a cross-sectional study, not a randomized study. The investigators were not blinded during data collection nor data analysis.

Reporting for specific materials, systems and methods

We require information from authors about some types of materials, experimental systems and methods used in many studies. Here, indicate whether each material, system or method listed is relevant to your study. If you are not sure if a list item applies to your research, read the appropriate section before selecting a response.

Materials & experimental systems

n/a	Involved in the study	
☒	☐	Antibodies
☒	☐	Eukaryotic cell lines
☒	☐	Palaeontology and archaeology
☒	☐	Animals and other organisms
☐	☒	Human research participants
☒	☐	Clinical data
☒	☐	Dual use research of concern

Methods

n/a	Involved in the study	
☒	☐	ChIP-seq
☒	☐	Flow cytometry
☒	☐	MRI-based neuroimaging

Human research participants

Policy information about studies involving human research participants

Population characteristics	A complete description of the study participants can be found in Tables S1 and S2.<br>We analysed a large set of available and newly sequenced metagenomic datasets (N=31) with known interpersonal relations, comprising 9,715 microbiome samples (7,646 stool and 2,069 saliva) and curated participant information. Eight cohorts (871 stool and 1,929 saliva samples) specifically targeted for microbiome transmission inference were sequenced in the context of this study, and another three 8,21 were further expanded (107 additional stool samples). Overall, human metagenomes from 20 different countries in five different continents and diverse lifestyles were included in the study.
Recruitment	All publicly available data that could be used to assess strain transmission (mother-infant, households, twin pairs) was included, limiting selection bias. Newly-included samples of existing datasets were collected and processed following the protocols described in the original publications. In addition, eight datasets (total: 2,800 samples) were newly collected and sequenced in the context of this study in collaboration with local partners, as detailed in the Methods section.
Ethics oversight	All study procedures are compliant with all relevant ethical regulations. The procedures were performed in compliance with the Declaration of Helsinki. Ethical approval of the Argentina cohort was granted by the Ethics and Safety committee (CEySTE), CCT Santa Fe, Argentina (29112019). The Colombia cohort was approved by the Research Bioethics committee, Universidad Metropolitana, Colombia (NIT 890105361-5). The China_1 dataset research protocol was approved by the Ethics Committee of Shanghai Tenth Hospital, Tongji University School of Medicine (SHSY-IEC-pap-18-1), and China_2 by the Ethics committee of the Health Science Center, Xi'an Jiaotong University, China (2016-114). The Guinea-Bissau study was approved by the Health Ethics National Committee (Comitê Nacional da Ética na Saude), Ministry of Public Health, Guinea-Bissau (076/CNES/INASA/2017) and by the London School of Hygiene and Tropical Medicine Ethics Committee (Reference Number 22898). The Italy_1 dataset research protocol was approved by the Ethics Committee of Santa Chiara Hospital, Trento, Italy (51082283 - 30/07/2014) and the Ethics Committee of the University of Trento, Italy, and Italy_2 by the Liguria Regional Ethics Committee, Italy (006/2019). Ethical approval for the USA dataset was granted by Western IRB (https://www.wcgirb.com/), with protocol tracking number WIRB20151664. Written informed consent was obtained from all adult participants, and from parents of underaged participants.

Note that full information on the approval of the study protocol must also be provided in the manuscript.
